# Chromosome and plasmid-borne P_LacO3O1_ promoters differ in sensitivity to critically low temperatures

**DOI:** 10.1038/s41598-019-39618-z

**Published:** 2019-03-14

**Authors:** Samuel M. D. Oliveira, Nadia S. M. Goncalves, Vinodh K. Kandavalli, Leonardo Martins, Ramakanth Neeli-Venkata, Jan Reyelt, Jose M. Fonseca, Jason Lloyd-Price, Harald Kranz, Andre S. Ribeiro

**Affiliations:** 1 Laboratory of Biosystem Dynamics and Multi-Scaled Biodata Analysis and Modelling Research Community, Faculty of Medicine and Health Technology, Tampere University, Korkeakoulunkatu 7, 33720 Tampere, Finland; 2 CA3 CTS/UNINOVA. Faculdade de Ciências e Tecnologia, Universidade Nova de Lisboa, Quinta da Torre, 2829-516 Caparica Portugal; 3 Gene Bridges, Im Neuenheimer Feld 584, 69120 Heidelberg, Germany; 4 Biostatistics Department, Harvard T.H. Chan School of Public Health, Boston, MA 02115 USA; 5 Infectious Disease and Microbiome Program, Broad Institute, Cambridge, MA 02142 USA

## Abstract

Temperature shifts trigger genome-wide changes in *Escherichia coli’*s gene expression. We studied if chromosome integration impacts on a gene’s sensitivity to these shifts, by comparing the single-RNA production kinetics of a P_LacO3O1_ promoter, when chromosomally-integrated and when single-copy plasmid-borne. At suboptimal temperatures their induction range, fold change, and response to decreasing temperatures are similar. At critically low temperatures, the chromosome-integrated promoter becomes weaker and noisier. Dissection of its initiation kinetics reveals longer lasting states preceding open complex formation, suggesting enhanced supercoiling buildup. Measurements with Gyrase and Topoisomerase I inhibitors suggest hindrance to escape supercoiling buildup at low temperatures. Consistently, similar phenomena occur in energy-depleted cells by DNP at 30 °C. Transient, critically-low temperatures have no long-term consequences, as raising temperature quickly restores transcription rates. We conclude that the chromosomally-integrated P_LacO3O1_ has higher sensitivity to low temperatures, due to longer-lasting super-coiled states. A lesser active, chromosome-integrated native *lac* is shown to be insensitive to Gyrase overexpression, even at critically low temperatures, indicating that the rate of escaping positive supercoiling buildup is temperature and transcription rate dependent. A genome-wide analysis supports this, since cold-shock genes exhibit atypical supercoiling-sensitivities. This phenomenon might partially explain the temperature-sensitivity of some transcriptional programs of *E*. *coli*.

## Introduction

*Escherichia coli* has evolved sophisticated regulatory programs to adapt to fluctuating environments that allow tuning gene expression so as to trigger appropriate responses^1,2^. In general, gene expression regulation occurs during transcription initiation^3^ and it can be performed, e.g., by transcription factors^4,5^, which act locally, affecting specific genes, and by σ factors^6–9^, which have more genome-wide effects.

Similarly, environmental changes can affect chromosomal DNA compaction, which is associated to supercoiling^10,11^ and is regulated by nucleoid associated proteins (NAPs)^12,13^. Interestingly, changes in DNA compaction has genome-wide effects^13–15^, causing the expression of some genes to increase while in others it decreases^4,16–18^.

DNA compaction and supercoiling have distinct effects on plasmid-borne and chromosome integrated genes (see e.g.^19^). One reason for this is that the chromosome has topologically constrained segments that allow supercoiling buildup^12,20–22^, as transcription occurs, since this process generates positive supercoiling ahead of the RNA polymerase (RNAP) and negative supercoiling behind it^23,24^. Meanwhile, plasmids lack discrete constraints. Thus, when positive and negative supercoiling emerge, they freely diffuse in opposite directions and annihilate each other^19^. Thus, in general, the transcriptional activity in plasmids is only affected by transient constraints due to, e.g., transient protein binding^19,25^. Exceptions are, e.g., plasmids encoding membrane-associated proteins that, by anchoring to the membrane^26–29^, can form longer lasting constraints. Other exceptions are plasmids carrying tandem copies of one or two DNA-binding sites^25,30^ and plasmids carrying the T7 promoter, when expressed in topA mutant strains^31^. Nevertheless, it is worth noting that *in vivo* measurements suggest that, prior to annihilation, transient supercoiling changes can influence transcription rates of both plasmid-borne and chromosomally-integrated promoters^31–33^.

Temperature shifts affect DNA supercoiling directly^34,35^ as well as indirectly, e.g., by affecting the interactivity between NAPs and chromosomal DNA^36^. This may explain why temperature down-shifts affect the activity of most chromosomal genes in *E*. *coli*^37^.

Another temperature-dependent event in transcription is promoter escape^38^, the stage at which the RNAP is freed from the promoter and moves downstream towards the elongation region of the DNA template^39^. The stronger the binding between the RNAP and the promoter, the longer it usually takes for the RNAP to escape the promoter and begin elongation^39^. One reason for this is that, for escape to succeed, the RNAP needs to pull a sufficient amount of downstream DNA into itself (so as to reach its active center), which involves breaking interactions between the RNAP and the promoter, and between the RNAP and initiation factors^38^, which are energy dependent processes.

Given the above, we hypothesized that plasmid-borne and chromosomally-integrated genes can differ in sensitivity to temperature shifts and that these differences may be promoter strength-dependent. To test this, we compared quantitatively the effects of temperature shifts on the *in vivo* kinetics of transcription of the P_LacO3O1_ promoter, when on a plasmid and when chromosomally-integrated (Materials and Methods). Further, we assessed the effects on the native *lac* promoter, whose transcription rate is weaker than P_LacO3O1_, although we located it in the same position in the chromosome.

For this, we used the MS2-GFP RNA tagging technique in *E*. *coli*, along with a recently proposed methodology to resolve the rate-limiting steps governing the *in vivo* dynamics of initiation of prokaryotic promoters (similar to established steady-state assays to resolve the *in vitro* dynamics)^40^. Further, we studied this process at critically low temperatures (below 23 °C), a regime in which most cellular processes exhibit significant differences due to, e.g., globally-altered transcription rates^37^ and increased cytoplasmic viscosity^41^. Using these techniques, we characterized, with single-RNA sensitivity, the RNA production dynamics of these constructs at various temperatures, as well in the presence of Gyrase and Topoisomerase I inhibitors and of DNP-based energy depletion. Also, we made use of stochastic modelling to show that the observed differences in transcription kinetics between chromosome and plasmid integrated promoters at low temperatures are consistent with current stochastic models of transcription initiation that account for supercoiling buildup, provided that such low temperatures result in the hindrance to escape from DNA super-coiling. Finally, we made use of information of what genes in *E*. *coli* have their activity induced following cold-shocks^42^ and of what genes are supercoiling sensitive^14^, to assess if these two features are strongly correlated, as our results would suggest.

## Results

We studied at the single-RNA level if the kinetics of RNA production under the control of P_LacO3O1_ differs in response to temperature changes when the gene is single-copy F-plasmid-borne and when it is chromosome-integrated. For this, we made use of two identical constructs under the control of the P_LacO3O1_ promoter coding for multiple bindings sites for MS2-GFP followed by the coding region of mCherry.

**Figure 1:**
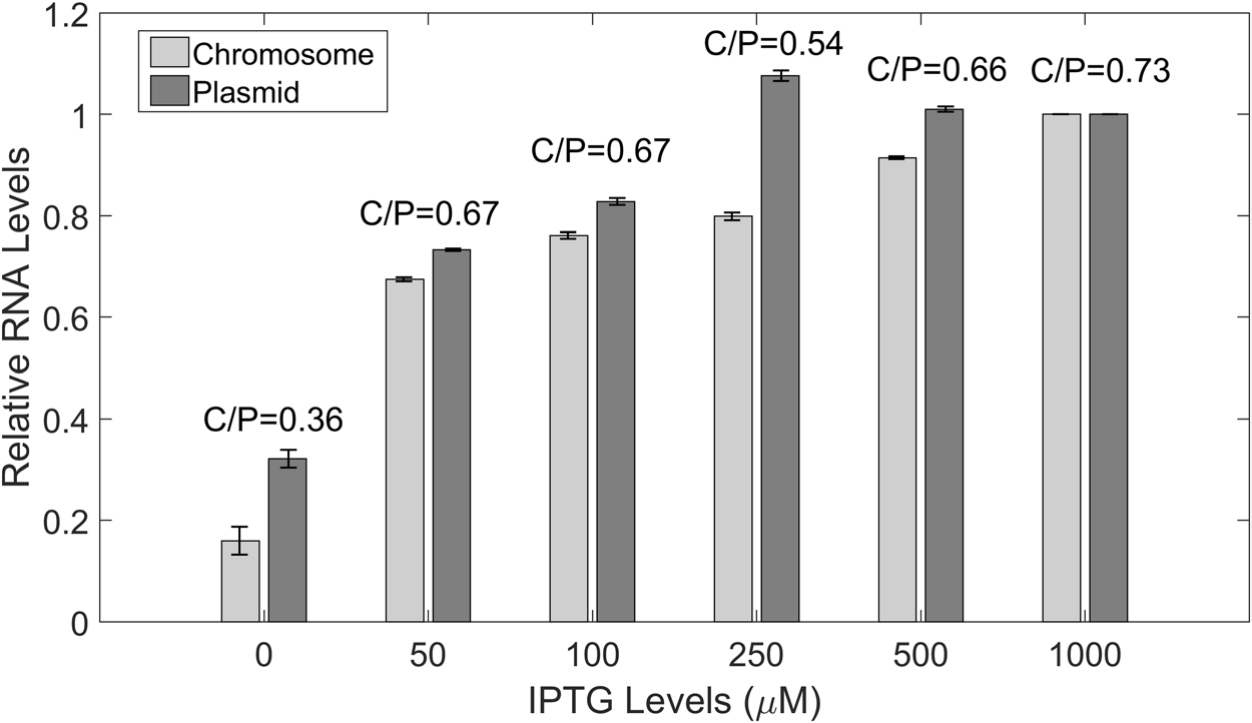
Induction curves, measured by microscopy imaging and single RNA tagging by MS2-GFP, of the target promoter P_LacO3O1_ when integrated into the chromosome (light grey) and into a single-copy F-plasmid (dark gray) (*E*. *coli* strain BW25993). Shown are the mean integer-valued RNA numbers (relative to the reference case, 1 mM IPTG) in individual cells of the two constructs, 1 hour after induction at 30 °C. Data presented as relative mean to the reference case with 90% confidence intervals obtained from a two-tailed Student’s t-test. Sample size per condition, as IPTG is increased, is (chromosome) 665, 655, 675, 670, 660 and 645 cells, and (plasmid) 670, 670, 665, 655, 655, 675 cells. Also shown is the ratio between the mean integer-valued RNA numbers per cell between cells with the target gene chromosome-integrated and on a single-copy plasmid. Absolute integer-valued RNA numbers per cell in each condition can be obtained from the absolute integer-valued RNA numbers per cell for 1 mM IPTG shown in Table 1 along with the relative values shown here. Results are obtained from 3 biological repeats. Since these exhibited no statistically significant differences, the results presented here are composed of the data from the 3 biological replicates.

Both constructs, shown in Figs S1 and S2, are functional and responsive to the inducer (Fig. 1). Also, control tests were performed to verify that spots detected in microscopy images correspond to MS2-GFP tagged RNA molecules (Fig. S3) and that, once appearing, their intensity does not change significantly during the measurement time (Supplementary section “Control tests of the RNA counting method”), as this would affect the counting of MS2-GFP tagged RNA molecules in each cell.

For each strain (carrying the target gene in the plasmid or in the chromosome) and each temperature condition, we performed 3 or more biological repeats and counted RNA-spots (MS2-GFP tagged RNAs) in each cell from the microscopy images (Supplementary section “Image Analysis”). As we did not find statistically significant differences between repeats, the results shown here are from cells from all replicates.

Since the two strains are not subject to the same antibiotics (only cells carrying the target gene in the single-copy plasmid are subject to chloramphenicol, see Methods), we tested whether their growth curves differ. Results in Fig. S5 show that these curves are not distinguishable. We also tested whether the two strains produce similar levels of MS2-GFP reporter proteins (as differences could result in different ability to count target RNAs). For this, inducing only the reporter gene, we measured the background fluorescence intensity of cells of both strains (almost exclusively due to MS2-GFP reporters). We then compared the two distributions of single-cell background fluorescence intensity by a Kolmogorov-Smirnov (KS) test of statistical significance and found no significant difference (p-value of 0.46).

### Induction of gene expression is similar, but not identical, in the chromosome and plasmid-integrated constructs

For each construct (chromosome integrated and single-copy plasmid borne) and IPTG concentration, we quantified the integer-valued RNA numbers in live cells at 30 °C by microscopy imaging, 1 hour after induction of the target promoter by IPTG (Materials and Methods). From Fig. 1, we find that the mean integer-valued RNA numbers per cell, relative to maximum induction (1 mM IPTG) exhibits a similar fold change in both constructs (~4 for the plasmid and ~5 for the chromosome construct), as in previous studies^43^. Also, for both constructs, the transcription rate does not increase beyond 500 µM IPTG, i.e., 1 mM IPTG suffices for full induction, in agreement with previous studies^40,43^.

However, the RNA production kinetics of the two constructs differs in some aspects. First, from Table 1 and Fig. 1, mean integer-valued RNA numbers per cell are higher in the plasmid construct for all induction conditions. Also, the increase in RNA numbers with IPTG concentration differs. Namely, from Table S3, in the plasmid construct the RNA numbers increase gradually as IPTG is increased, allowing most conditions to differ significantly in a statistical sense, while in the chromosome construct the RNA numbers per cell only differ significantly between 0 µM IPTG and the other conditions (for 50 µM IPTG or higher, there is little increase in RNA numbers with additional increases in IPTG concentrations).

**Table 1:** Number of cells observed, mean, and squared coefficient of variation (CV^2^) of the absolute integer-valued RNA numbers per cell for the chromosome-integrated and the plasmid-integrated constructs, when induced by 1 mM IPTG.

Condition	No. cells	Mean integer-valued RNA no. per cell	CV^2^
**Chromosome construct**			
30 °C	645	2.08	2.60
27 °C	632	2.00	1.93
23 °C	668	1.74	2.30
20 °C	646	0.59	7.17
16 °C	668	0.22	15.81
10 °C	648	0.25	16.12
**Plasmid construct**			
30 °C	675	2.86	1.06
27 °C	654	2.46	1.42
23 °C	665	1.63	2.73
20 °C	660	1.61	3.08
16 °C	663	1.50	2.99
10 °C	676	1.35	3.46

Cells are induced and kept at 30 °C, 27 °C, 23 °C, 20 °C, 16 °C and 10 °C for 60 minutes prior to the acquisition of the results. Results are obtained from 3 biological repeats. Since these exhibited no statistically significant differences, the results presented here are composed of the data from the 3 biological replicates.

Nevertheless, the same model of transcription (Supplementary Information, section “Model of transcription kinetics”, reactions 1–3) fits both constructs, as tuning k_cc_ and/or k_unlock_ suffices to account for differences between them in RNA numbers at 30 °C.

### Transcription by the chromosome-integrated construct is noisier at lower temperatures

We next studied if temperature changes affect differently the chromosome and plasmid constructs. We measured integer-valued RNA numbers in cells under full induction (1 mM IPTG) by microscopy at various temperatures (30, 27, 23, 20, 16 and 10 °C). For each condition, from the absolute integer-valued RNA numbers in each cell, we calculated the mean and squared coefficient of variation (CV^2^) of the RNA numbers in single cells (Table 1). To assess if the RNA production kinetics differs with temperature and between the two constructs, we performed KS tests. The P values comparing the single-cell distributions of RNA numbers between conditions for the chromosome and plasmid constructs and between the constructs at each temperature are shown in Tables S4 and S5, respectively.

From Tables 1 and S4, we find that P_LacO3O1_, when in the single-copy plasmid, is highly responsive to temperature decreases until 23 °C. Below this temperatures, changes in RNA numbers are only significant for temperature shifts wider than those considered in Table S4 (e.g. p < 0.01 for 23 °C and 10 °C, not shown in Table S4). This behavior is in line with previous reports for the P_TetA_ and for the P_Lac-Ara-1_ promoters, also on single-copy plasmids^44^.

Meanwhile, when chromosome-integrated, P_LacO3O1_ activity decreases significantly for a wider range of temperatures. Namely, differences are detectable between all pairs of neighboring conditions, except between 16 °C and 10 °C. These results are supported by those in Table S5. The P values of the KS tests indicate that, below 23 °C, the plasmid and chromosome constructs differ from one another in all temperatures. For 23 °C and above, they only differ at 30 °C. From this and Table 1, we conclude that the activity of the chromosome-integrated promoter is more heavily reduced as temperature is lowered, and that it remains sensitive to a wider range of temperature shifts.

To validate these results, we used RT-qPCR (Supplementary Information) to obtain the mean RNA numbers relative to the control (30 °C) in cells under full induction (1 mM of IPTG) at 23, 16 and 10 °C (Fig. S6). From Table 1, we calculated the same quantities from the microscopy measurements. Overall, both the chromosome and plasmid constructs exhibit the same qualitative behavior as temperature decreases when measured by microscopy and RT-qPCR.

We next assessed if the weaker transcriptional activity of the chromosome-integrated promoter at the lowest temperatures could be explained by changes in the spatial distribution of RNAPs^45^ or of the nucleoids (Supplementary Information, section “Nucleoid staining with DAPI”). Measurements at 10 °C and 30 °C (Fig. S7) show no significant differences in these two features, allowing rejecting these hypotheses.

**Figure 2:**
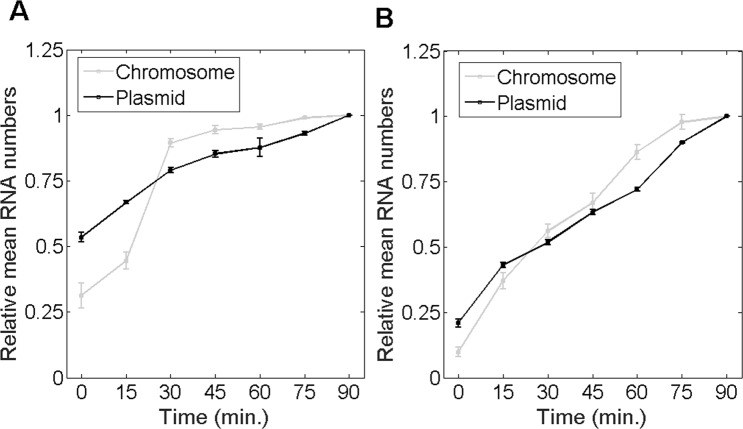
Mean integer-valued RNA numbers in individual cells, relative to the last time moment, as a function of temperature, measured by microscopy with single RNA tagging by MS2-GFP, when P_LacO3O1_ is integrated into the chromosome (light grey) and in a single-copy F-plasmid (dark gray). (**A**) Cells are at 10 °C. (**B**) Cells are at 30 °C. Data presented as relative mean to the reference case with 90% confidence intervals obtained from a two-tailed Student’s t-test. Sample size per condition, as time progresses is: (**A**) Chromosome at 10 °C (610, 611, 615, 610, 609, 602 and 605 cells), and Plasmid at 10 °C (615, 610, 610, 612, 608, 606 and 606 cells). (**B**) Chromosome at 30 °C (604, 615, 610, 610, 605, 608 and 605 cells), and Plasmid at 30 °C (610, 615, 606, 615, 610, 613 and 609 cells). For each time point, new cells were taken from the original culture. Results are obtained from 3 biological repeats. Since these exhibited no statistically significant differences, the results presented here are composed of the data from the 3 biological replicates. Finally, at t = 0 min, the mean absolute number of RNA molecules per cell was (**A**) 0.1 for chromosome and 0.9 for plasmid, and (**B**) 0.2 for the chromosome and 0.9 for the plasmid.

We also performed two additional tests for cells with the chromosome-integrated P_LacO3O1_. First, as the mean integer-valued RNA numbers per cell in induced cells at 10 °C (Table 1) appears to be smaller than in non-induced cells at 30 °C (Table S1), we tested if this difference is statistically significant by performing a KS test between the distributions of single-cell RNA numbers in the two conditions. We obtained a p-value of 0.99 and, thus, we conclude that the RNA numbers in the two conditions do not differ, in a statistical sense (p-value larger than 0.01), implying that induced cells at 10 °C produce at least as much RNAs as non-induced cells at 30 °C. Second, we tested whether, at 10 °C, RNA numbers differ between induced and non-induced cells. A KS test between the distributions of RNA numbers in individual cells in the two conditions (Fig. 2 and Table S1) shows that they can be distinguished in a statistical sense. Thus, we concluded that induction at 10 °C tangibly increases the RNA production rate of the chromosome-integrated P_LacO3O1_.

### Mean relative time prior to commitment to transcription increases in the chromosome-integrated construct at low temperature

To investigate why the two constructs responded differently to lowering temperatures, we assessed whether the changes in the kinetics of transcription with decreasing temperature occur *prior to* or *following* the commitment to open complex formation (Supplementary Information, section “Model of transcription kinetics”), by making use of Lineweaver–Burk plots^46^ of the inverse of the RNA production rate against the inverse of the RNAP concentration (Supplementary sections “Lineweaver-Burk Plots” and “Tuning intracellular RNAP concentrations”).

For this, we measured RNAP levels in individual cells in each temperature condition, and verified that they differ statistically between conditions (KS-tests in Table S8). From these, we obtained the inverse of the RNAP concentrations relative to the 1X control condition (Table S7). Next, for the same condition, using the region of the target gene coding for mCherry (Fig. S2), we measured the RNA production rates of the two constructs at 10 and 30 °C by RT-qPCR, and obtained the inverse of these values (Table S9). Combining both measurements, we obtained Lineweaver–Burk plots for each construct and the two temperature conditions (example Fig. S10).

Next, from these plots, using the same methodology as in^40,43,47^, we estimated the mean fraction of time between consecutive transcription events taken by the steps preceding $(\frac{{t}_{prior}}{{\rm{\Delta }}t})$ and following $(\frac{{t}_{after}}{{\rm{\Delta }}t})$ the commitment to open complex formation^40,43^ at the highest and lowest temperature, with Δt being the mean time length between consecutive transcription events in individual cells (Supplementary Information, section “Relative mean duration prior to and following commitment to transcription”).

Results in Table 2 show that, in all 4 conditions, the most rate-limiting events occur after commitment to open complex formation. However, Table 2 also informs that the lowering temperatures do not cause the same effect in the two constructs.

**Table 2:** Relative mean duration of the rate limiting steps in transcription initiation at 30 °C and 10 °C.

Condition	$\frac{{{\boldsymbol{t}}}_{{\boldsymbol{p}}{\boldsymbol{r}}{\boldsymbol{i}}{\boldsymbol{o}}{\boldsymbol{r}}}}{{\boldsymbol{\Delta }}{\boldsymbol{t}}}$	$\frac{{{\boldsymbol{t}}}_{{\boldsymbol{a}}{\boldsymbol{f}}{\boldsymbol{t}}{\boldsymbol{e}}{\boldsymbol{r}}}}{{\boldsymbol{\Delta }}{\boldsymbol{t}}}$
**30 °C**		
Chromosome construct	0.09	0.91
Plasmid construct	0.08	0.92
**10 °C**		
Chromosome construct	0.27	0.73
Plasmid construct	0.02	0.98

Shown are the mean durations, relative to the mean time-length of the intervals between transcription events (∆t), of the rate-limiting steps prior $(\frac{{t}_{prior}}{{\rm{\Delta }}t})$ and after $(\frac{{t}_{after}}{{\rm{\Delta }}t})$ commitment to open complex formation for the chromosome-integrated and the plasmid-integrated constructs.

In particular, in the plasmid construct, in agreement with previous *in vitro* measurements for the synthetic P_Lac-UV5_ promoter^4^, the reduction in RNA production rate with lowering temperature is mostly due to a reduction in the rate of the events *after* commitment to open complex formation (with *t*_*after*_ increasing from being 92% to 98% of the Δt as temperature is lowered). Meanwhile, in the chromosome construct, the opposite occurs (with *t*_*after*_ decreasing from 91% to 73% of the Δt as temperature is lowered) suggesting that, in this construct, the events whose rates were most reduced occur prior to commitment to open complex formation, provided that the RNA production rate decreases with lowering temperature (as is the case, see Table 1).

### Local DNA supercoiling in the chromosomally-integrated gene drives the differences between constructs

To explain the increased time-length of the events preceding the open complex formation in the chromosome-integrated construct at lower temperatures, we considered the model of transcription initiation (Supplementary Information, reactions 1–3). This model allows for this, provided that decreasing temperatures decrease the rate of unlocking (k_unlock_) from locked promoter states (reaction 3, Supplementary Information), or decrease the rate of unbinding of a repressor from a promoter (k_ON_, reaction 2 in Supplementary Information), or both. Either of these possibilities is physically possible since lowering temperatures could affect the efficiency of repressors (see e.g.^48^), DNA packaging (known to differ between plasmid and chromosomes^49^, or DNA super-coiling^35^ (known to affect both packaging^10^ and transcription^19,30,50,51^).

A third possibility would be that decreasing temperature modified the kinetics of closed complex formation, causing increased relative duration of this event, e.g. due to reduced k_1_ or k_2_, or instead increased k_−1_. However, this would result in reduced noise in RNA production^40,52^ and thus reduced CV^2^ in RNA numbers in individual cells (since, at 30 °C, most time between transcription events is spent in open complex formation, Table 2). The data on CV^2^ in RNA numbers in Table 1 disproves this possibility.

Meanwhile, in the first possibility, where k_unlock_ or k_ON_ are decreased with decreasing temperature, this would result in increased noise in RNA production^53^ and, thus, increased CV^2^ in RNA numbers in individual cells, which was observed (Table 1).

To determine whether it is k_unlock_ or k_ON_ that is decreased, consider that a change in repressors’ efficiency with temperature (i.e. a change in k_ON_) should affect both the chromosome and plasmid constructs similarly since both constructs are affected by this mechanism. However, we observed divergent responses between these two constructs to the lowest temperatures, with the plasmid-borne construct being unable to turn off its RNA production as efficiently as the chromosome-integrated construct (Table 1). Thus, we conclude that the stronger decrease in the chromosome construct in RNA production rate with lowering temperature (at the lowest temperature conditions tested) is likely due to an increased amount of time required to remove the promoter from the locked state, which does not occur in the plasmid construct (i.e. changes in k_unlock_ with lowering temperature are the most likely explanation for the observed behaviors).

It is further possible to assess if the changes in k_unlock_, causing different behaviors of the two constructs in response to lowering temperatures, are associated to DNA packaging and/or super-coiling. For that, we measured the nucleoid size in cells with one nucleoid (Supplementary Information) in the various temperature conditions. If decreasing temperature (in the ranges shown in Table 1) affects DNA packaging significantly, we expect differences in the mean and/or variability of the nucleoid size. However, we found no significant differences between 10 °C and 30 °C (Table S10). Similar results were reported in^41^. Thus, we discard DNA packaging as the main cause for the differences between chromosome and plasmid response to temperature shifts.

Given all of the above, we hypothesize that the difference in response of the chromosome and plasmid-integrated genes with lowering temperature is due to an increased rate of accumulation of local DNA supercoiling in the chromosome-integrated gene, which increases the escape times from locked states (reactions 1 and 3, Supplementary Information).

To validate this hypothesis, we performed several experiments. First, we compared the numbers of RNAs produced over time by cells of each strain. We expect this number to increase near-constantly in the cells carrying the plasmid-borne gene, but not in the cells of the other strain. For this, from the moment of activation of the target gene (t = 0 minutes), we measured integer-valued RNA numbers in individual cells at 10 °C every 15 minutes for 90 minutes (for each time point, new cells were taken from the original culture). If the weaker activity of the chromosome-integrated promoter is due to increased propensity to be in the locked state due to the accumulation of DNA super-coiling, we expect its transcription activity to be blocked after a few events. At a population level, this would result in a sharp decrease in the rate of increase of RNA numbers in the cells, some time after the start of the measurements. Meanwhile, in the plasmid construct, we expect a constant RNA production rate over time, due to the lack of accumulation of local DNA super-coiling^19^. Results in Fig. 2A confirm these predictions. Cells at 10 °C with the chromosome construct only exhibit production in the first 30 minutes, while the plasmid construct shows approximately constant RNA production rate throughout the measurement.

We also performed measurements at 30 °C. Given the similar dynamics of transcription of the two constructs in this condition (Table 2), we expect the RNA production rate to be constant in time in both constructs. Results in Fig. 2B confirm this.

**Figure 3:**
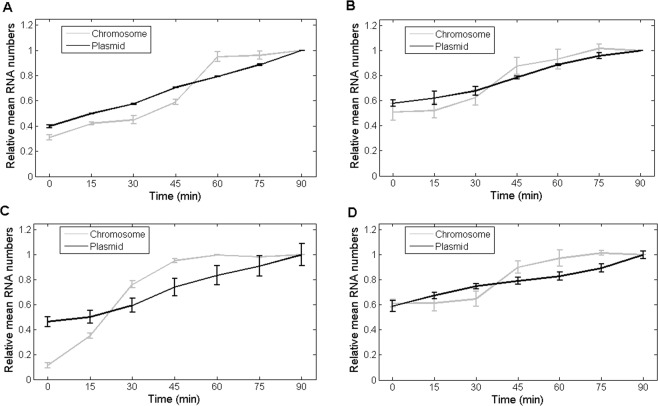
Mean integer-valued RNA numbers in individual cells, relative to the last time moment, as a function of temperature and gyrases and topoisomerases I inhibitors, measured by microscopy with single RNA tagging by MS2-GFP, when P_LacO3O1_ is integrated into the chromosome (light grey) and in a single-copy F-plasmid (dark gray). (**A**) Cells at 30 °C and subject to Novobiocin. (**B**) Cells at 10 °C and subject to Novobiocin. (**C**) Cells at 30 °C and subject to Topotecan. (**D**) Cells at 10 °C and subject to Topotecan. Data presented as relative mean to the reference case with 90% confidence intervals obtained from a two-tailed Student’s t-test. Sample size per condition, as time progresses is: (**A**) Chromosome, 30 °C, Novobiocin (608, 606, 605, 610, 613, 610 and 615 cells) and Plasmid, 30 °C, Novobiocin (615, 605, 613, 610, 610, 601 and 602 cells); (**B**) Chromosome, 10 °C, Novobiocin (615, 610, 615, 625, 605, 620 and 620), and Plasmid, 10 °C, Novobiocin (615, 620, 615, 610, 620, 620 and 615 cells); (**C**) Chromosome, 30 °C, Topotecan (615, 610, 612, 610, 605, 603 and 610 cells) and Plasmid, 30 °C, Topotecan (662, 623, 626, 606, 643, 659 and 647 cells) and, finally, (**D**) Chromosome, 10 °C, Topotecan (620, 610, 620, 610, 610, 610 and 615 cells) and Plasmid, 10 °C, Topotecan (679, 629, 649, 645, 642, 601 and 632 cells). For each time point, new cells were taken from the original culture. Results are obtained from 3 biological repeats. Since these exhibited no statistically significant differences, the results presented here are composed of the data from the 3 biological replicates. In all cases, Novobiocin or Topotecan was added to the culture at the same time as the inducer of the target gene, IPTG. Finally, at t = 0 min, the mean absolute number of RNA molecules per cell was (**A**) 0.3 for chromosome and 0.9 for plasmid, (**B**) 0.1 for chromosome and 0.9 for plasmid, (**C**) 0.1 for chromosome and 0.9 for plasmid, and (**D**) 0.1 for chromosome and 0.9 for plasmid.

To further test the hypothesis, we next compared the activity of the two constructs at 30 °C when subjecting cells to Novobiocin, an inhibitor of Gyrase activity (Methods)^19,54^. Gyrase releases positive supercoiling^55^ but not negative supercoiling^56^. According to the twin-supercoiled-domain model^24^, which predicts that negative/positive supercoils should accumulate in the absence of supercoil-relaxing enzymes, we expect cells with the chromosome construct to exhibit a similar behavior as when at 10 °C. Meanwhile, cells with the plasmid construct should again exhibit a constant rate of transcription over time^19^. Figure 3A confirms these predictions.

In this regard, in both strains, the gene *acrA* is present, and thus, Novobiocin is not expected to affect cell division rates^57^. To test this, we measured cell growth rates by OD_600_ for varying Novobiocin concentrations (0, 50, 75, 100 and 150 ng/μl). We found the growth rates to not differ significantly between conditions (data not shown). These results also show that 100 ng/μl Novobiocin concentration suffices to affect (but not halt) the transcription rate of the chromosome-integrated gene (compare the results for this construct in Figs 2B and 3A, at 30 °C).

Subsequently, we subject cells with the chromosome construct to Novobiocin when at 10 °C. Results in Fig. 3B, when compared to Figs 2A and 3A, show that transcription in cells carrying the chromosome is more strongly blocked when combining Novobiocin and low temperatures. I.e. while at 10 °C alone and subject to Novobiocin alone, RNA numbers increase by a factor of 4 (from 0.25 to 1) in a period of 90 min., when subjecting cells to both 10 °C and Novobiocin, the RNA numbers increase only by a factor of 2 (from 0.5 to 1) in the same period of time. Meanwhile, in cells with the plasmid construct, we observe the same RNA production as in Fig. 2A, meaning that, in these cells, Novobiocin has no effect at either temperature. Given this, we suggest that the transcription activity of the chromosome-integrated promoter at 10 °C is hampered by an increased difficulty in unblocking the DNA from supercoiled states that is not due to a loss of functionality of Gyrases.

Then, we observed cells with the chromosome integrated gene at 30 °C, when subject to Topotecan, an inhibitor of Topoisomerase I activity^58,59^ (Methods). Topoisomerase I releases negative, but not positive supercoiling^60^. We expect these cells to exhibit a similar behavior as when at 10 °C, which Fig. 3C confirms. Also, we observed the same cells subject to Topotecan when at 10 °C. From Fig. 3D, transcription is again blocked more strongly than when at 10 °C but not subject to Topotecan and when at 30 °C subject to Topotecan. Namely, while in the latter two conditions RNA numbers increased by a factor of 4 (from 0.25 to 1) in 90 min., when subjecting cells to both 10 °C and Topotecan the RNA numbers increase only by a factor of 2 (from 0.5 to 1) in the same period of time.

These results suggest that the activity of the chromosome-integrated gene at 10 °C is hampered by an increased difficulty in unblocking the DNA from supercoiled states, rather than due to a loss of functionality of Topoisomerases I (or Gyrases). Table S15, with the results of the KS tests between the distributions of RNA numbers in individual cells at 10 °C and 30 °C, when subject to Novobiocin or Topotecan, confirm that the distributions differ with temperature, in a statistical sense.

Finally, in comparison, subjecting cells with the single-copy F-plasmid to Topotecan causes, qualitatively, the same behavior as adding Novobiocin (at 30 °C and 10 °C) (Fig. 3A–D).

### Promoter escape from supercoiling buildup is similarly hampered if cellular energy is depleted

If the escape from DNA supercoiling buildup in the chromosomally-integrated construct at low temperatures is due to energy deficiency at low temperatures (the energy required for the necessary endothermic reactions to occur should be higher in such conditions), it should be possible to mimic the phenomena by, instead of lowering temperature, depleting cells of energy via DNP treatment^45^ (Methods). In particular, we expect cells subject to this treatment to, even at 30 °C, be less able to maintain the chromosome integrated promoter active over time when compared to the control, similar to when at 10 °C.

**Figure 4:**
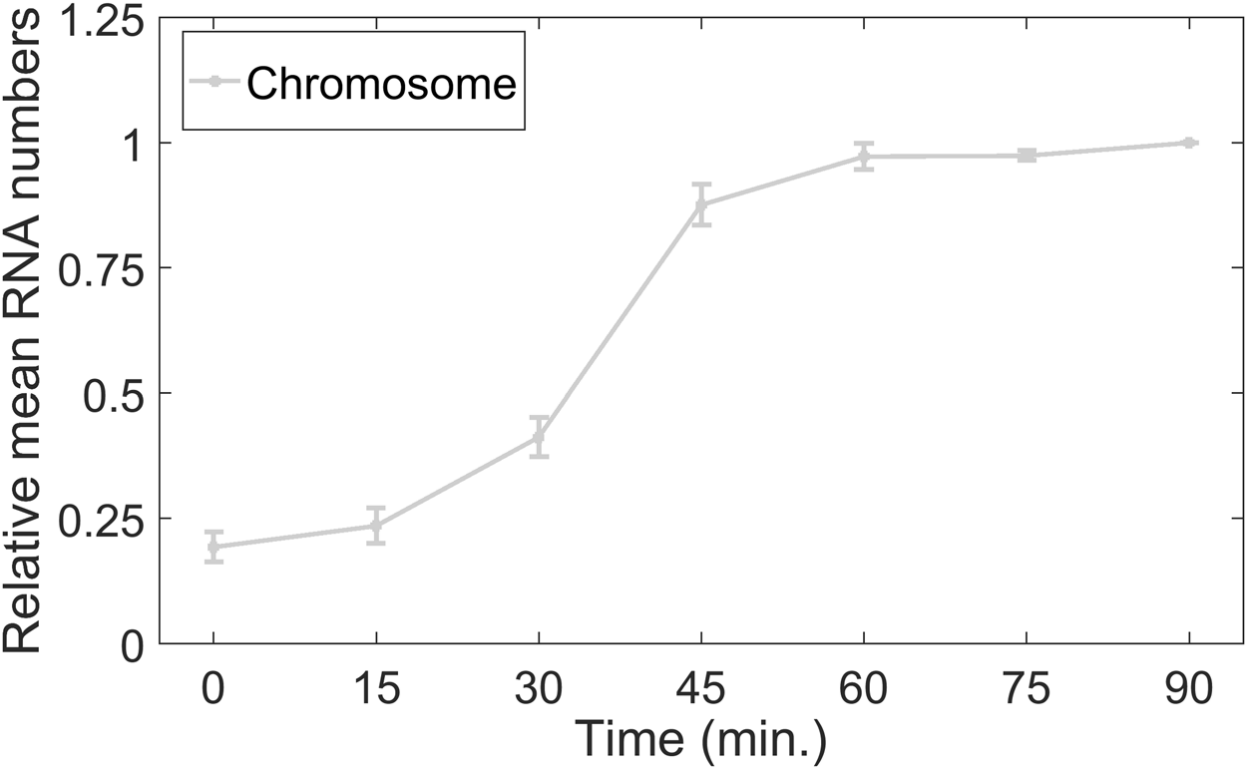
Mean integer-valued RNA numbers in individual cells at 30 °C subject to DNP treatment relative to the last time moment, measured by microscopy with single RNA tagging by MS2-GFP, when P_LacO3O1_ is integrated into the chromosome (light grey). Data presented as relative mean to the reference case with 90% confidence intervals obtained from a two-tailed Student’s t-test. Sample size per condition, as time progresses is 601, 610, 601, 605, 610, 605 and 608 cells. For each time point, new cells were taken from the original culture. Results are obtained from 3 biological repeats. Since these exhibited no statistically significant differences, the results presented here are composed of the data from the 3 biological replicates. Finally, at t = 0 min, the mean absolute number of RNA molecules per cell was 0.2.

To test this, we subjected cells to DNP for 90 minutes (at 30 °C) prior to imaging (Methods). As expected, we observed similar RNA production dynamics (Fig. 4), as in untreated cells at 10 °C with a chromosome integrated P_LacO3O1_ (Fig. 2A). I.e., beyond 30 minutes, there is little to no transcription, suggesting that, in this condition, the activity is also being hampered by increased difficulty in escaping from supercoiled states.

### Low temperatures have no long-term consequences on transcription blocking by DNA supercoiling

We performed an additional test to support the hypothesis that the escape from DNA supercoiling buildup in the chromosome construct at low temperatures is due to energy deficiency. Namely, we hypothesized that changing temperature to near-optimal conditions (e.g. 30 °C) should restore the cells’ ability to relax DNA supercoiling (as at higher temperature this process is expected to require less energy). To test this, we subjected cells with a chromosome integrated P_LacO3O1_ to two temperature shifts, first from high (30 °C) to low (10 °C) and then from low (10 °C) to high (30 °C), and measured the mean integer-valued RNA numbers in the cells over time.

**Figure 5:**
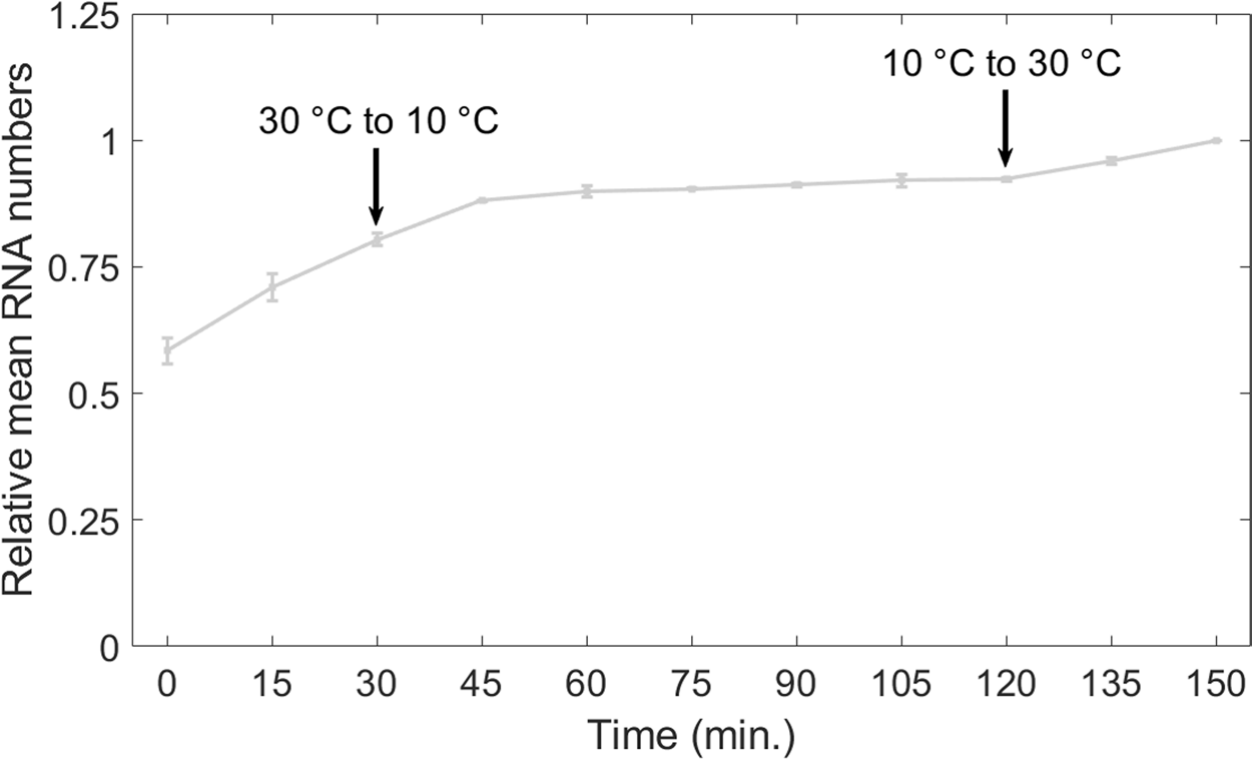
Mean RNA numbers in individual cells, relative to the last time moment, as a function of temperature shifts, measured by microscopy with single RNA tagging by MS2-GFP, when P_LacO3O1_ is integrated into the chromosome (light grey). In these measurements of integer-valued RNA numbers of P_LacO3O1_’s activity when integrated in the chromosome, first, the cells are kept at 30 °C for 30 minutes. Next, they are kept at 30 °C and measurements are conducted (starting point of the measurements is defined as moment t = 0). 30 minutes after starting the measurements, the temperature is changed to 10 °C and then kept constant until reaching moment 120 min. Then it is altered again to 30 °C and kept constant until the end of the measurements. Data presented as relative mean to the reference case with 90% confidence intervals obtained from a two-tailed Student’s t-test. Sample size per condition, as time progresses is 600, 601, 603, 615, 613, 610, 603, 614, 611, 608, 607 cells. For each time point, new cells were taken from the original culture. Results are obtained from 3 biological repeats. Since these exhibited no statistically significant differences, the results presented here are composed of the data from the 3 biological replicates. Finally, at t = 0 min, the mean absolute number of RNA molecules per cell was 1.3.

Results in Fig. 5 show that both temperature shifts caused smooth transitions in the RNA production rates that are consistent with changes in the kinetics of locking/unlocking of promoters from positive supercoiling buildup. In detail, cells at 30 °C have constant RNA production, as shown previously. Once temperature is shifted to 10 °C, after 15–30 minutes, little to no RNA production is observed (as in Fig. 2A). More importantly, once high temperatures are restored (to 30 °C), RNA production is quickly restored to nearly the original rate. The fast transition between behaviors and the ability to quickly restore the original dynamics reinforce the conclusion that the activity of the chromosome integrated promoter at 10 °C is hampered by an increased difficulty in unblocking the promoter from supercoiled states (due to an increase in the energy required).

Note that in this particular experiment, following the shift from 30 °C to 10 °C, it does not follow a transient of ~15–30 minutes of reduced transcription activity that is visible in Figs 2, 3 and 4. This is because, in this case, when the shift occurs, the cells already contain sufficient IPTG to achieve full transcription rates, while in the previous experiments the inducer was added immediately before the microscopy measurements began, and thus, a transient time to reach quasi-equilibrium RNA production rates is expected, due to the non-negligible time that cells need to intake inducers from the media^38^, particularly at low temperatures. In the case of IPTG, previous measurements suggest that this transient is ~15–30 minutes long^61^, in agreement with the results in Figs 2A, 3A–D and 4.

### Stochastic modelling also suggests increased long-lasting super-coiled states at critically low temperatures to be the cause for enhanced sensitivity to shifts to critically low temperatures

We tested whether the increase in the expected time for promoters to escape from a supercoiling state across the cell population is, in accordance with current stochastic models of transcription in *E*. *coli*^40,43^ a plausible explanation for the change with decreasing temperature in the average RNA numbers over time in cells with the chromosome integrated promoter (Fig. 2). For this, we use the stochastic model of transcription initiation (Supplementary Information, reactions 1–3), derived from multiple studies, including genome-wide studies of variability in transcript counts^62,63^ and studies of the transcription dynamics of individual genes^40,43^.

All parameter values (Table S11) are from single-cell, single-RNA empirical data on the activity of *lac* derivative promoters^19,40^. Mean RNAP numbers are set to correspond to the RNAp concentration reported in^40^. Finally, from the results above, we assume that the increase in $(\frac{{t}_{prior}}{{\rm{\Delta }}t})$ as temperature decreases (Table 2) is mostly due to a decrease in k_unlock_. Thus, the remaining rate constants are, for simplicity, unchanged.

For each value of k_unlock_ tested, we performed 500 independent simulations, each 75 minutes long. Data was collected every 15 minutes, as in the experiments (Fig. 2). The values of k_unlock_ were selected as follows: the highest value, corresponding to high temperatures (30 °C), is reported in^19^. This value was then gradually lowered until the mean number of RNAs per cell at the end of the measurement period was similar to that observed in cells at 10 °C.

We assessed if the model was able to reproduce the observed RNA numbers over time at both high and low temperatures, and if there is a gradual behavioral change between these extreme conditions. For this, the initial numbers of all molecular species were set to zero, with the exception of P_ON_ (set to 1, corresponding to one active promoter per cell), RNAp (as noted above), and RNA. Initial RNA numbers were drawn randomly from a Poisson distribution (0.7 RNA/cell) to match the here observed outcome of spurious RNA production events. We observed also (empirically, Supplementary Table S1) that this number did not differ with temperature, as expected, since, prior to moment 0, cells were at the same temperature (30 °C) in both measurements.

**Figure 6:**
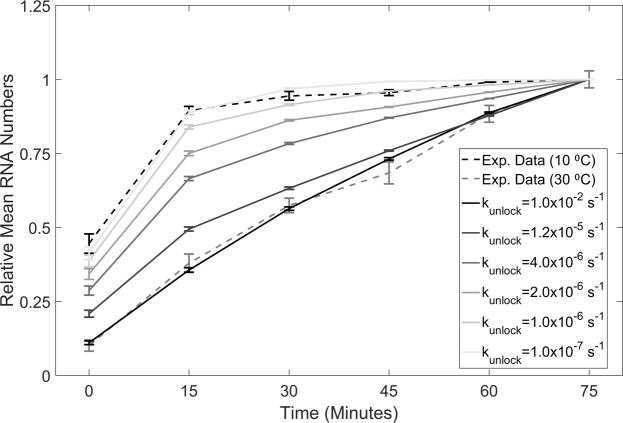
Expected mean RNA numbers in individual cells, relative to the last time moment, from simulations of a stochastic transcription model. The model assumes that the promoter is integrated into the chromosome for differing values of the rate of promoter escape from the supercoiled state (solid lines increasingly darker as k_unlock_ increases). Also shown are the measurements of mean RNA numbers from cells at 10 °C (dashed black line) and cells at 30 °C (dashed grey line). Data presented as relative mean to the reference case with 90% confidence intervals obtained from a two-tailed Student’s t-test.

In Fig. 6, we compared the results of the model with those in Fig. 2A (10 °C) and Fig. 2B (30 °C) for the chromosome-integrated promoter. For simplicity, as noted, we ignored the first time moment of the empirical data (0 minutes following induction) since, at this stage, the cells did not yet have fully active transcription^61^. This removed the need to model the intake process for the inducers^61^.

Results in Fig. 6 support the earlier conclusions. The accuracy with which the model reproduces the measurements suggests that the difference in mean RNA production rates between cells with the chromosome-integrated promoter at critically low (10 °C) and at high (30 °C) temperatures can be explained by a reduced ability to release chromosome-integrated promoters from the effects of DNA supercoiling at critically low temperatures.

Finally, note that setting k_unlock_ to infinite in reaction 3 (equivalent to having a model that does not allow promoter locking) results in a similar behavior to that of the plasmid-borne construct, and thus to the chromosome integrated promoter at 30 °C (data not shown).

### Transcription by a lesser active chromosome-integrated promoter construct is less sensitive to gyrase overexpression and temperature shifts

The influence of positive supercoiling buildup on the dynamics of a chromosome-integrated gene differs with its location on the chromosome due to, among other, differences in the expression rates of operons in different DNA loops^64^, which will cause the effects of positive supercoiling buildup to differ. Meanwhile, we observed that temperature downshifts have weaker effects on the plasmid-borne P_LacO3O1_ than on the chromosome-integrated P_LacO3O1_, since the latter is affected by positive supercoiling buildup. This implies that temperature down-shifts affect the various steps in transcription initiation by different degrees (as suggested in^44^), with escape from positive supercoiling buildup being one of the most affected.

Further, from above, it is reasonable to hypothesize that the effects of temperature downshifts on RNA production rates may be positively correlated with the expression rate of the chromosome-integrated promoter of interest. Namely, consider that the expected time for a Gyrase to intervene is determined, among other, by its intracellular concentration. As such, when increasing the transcription rate of a gene, it should become less likely for Gyrase to remove positive supercoiling buildup between transcription events. Similarly, one can also hypothesize that a chromosome-integrated promoter similar in functioning to, but with weaker activity than P_LacO3O1_, should be *relatively* less sensitive to temperature downshifts. I.e., its RNA production rate should be less reduced by a temperature downshift, relative to the control condition (i.e. more similar in behavior to the plasmid-integrated P_LacO3O1_).

To test the first hypothesis, we compared the effects of overexpressing Gyrase (Methods) on the mean RNA numbers in cells with the chromosome integrated promoter at 10 °C, when and when not induced. When induced (1 mmM IPTG), we find that overexpressing Gyrase increases the mean number of RNAs per cell by 154%. Meanwhile, when not induced (0 mmM IPTG), this mean number only increases by 107%. This agrees with the hypothesis that, the higher the expression rate, the bigger is the impact of lowering temperatures due to decreased probability that Gyrases can act in between transcription events.

To test the second hypothesis, we replaced the P_LacO3O1_ chromosome-integrated promoter by the native *lac*, which has a similar repression-activation mechanism (Methods) but weaker activity^65^. First, to confirm that its transcription rate is weaker than P_LacO3O1_, we measured the mean number of RNA molecules per cell, 1 hour after induction, at 30 °C and at 10 °C, under full induction. We found that the native *lac* produces ~85% less RNAs than P_LacO3O1_ (both at 30 °C and at 10 °C). Next, to test the hypothesis, we compared the effects of overexpressing Gyrase in cells at 10 °C, when carrying the chromosome-integrated P_LacO3O1_ and when carrying the chromosome-integrated native *lac*. Given the above, we expect the overexpression of Gyrase to have a weaker impact on the transcription rate of the native *lac*. Results in Table S16 confirm this, showing that the overexpression of Gyrase does not significantly affect its number of transcripts, similarly to the plasmid-borne P_LacO3O1_. Meanwhile, measurements at 30 °C showed, as expected, that no construct is significantly affected by overexpressing Gyrase (Table S16).

### Cold shock-inducible genes have atypical sensitivity to supercoiling

Given the above results that show that temperature downshifts can affect the escape times from positive supercoiling buildup, one can hypothesize that genes essential for adaptation to low temperatures (e.g. cold shock inducible genes) should have atypical sensitivity to supercoiling buildup. I.e., these genes should be either significantly more responsive or significantly less responsive to supercoiling buildup than randomly selected genes.

To test this, we combined information on *E*. *coli*’s genome-wide sensitivity to supercoiling buildup (enhanced by repressing Gyrase)^14^, with information on genome-wide responsiveness to cold shocks^42^. Cold-shock responsive genes were classified as being associated to transient or to prolonged responses^42^. We find that the genes responsible for transient responses (70 genes) are two times more likely (~14% chances) to be supercoiling sensitive than a gene selected randomly from the genome (~7% chances, since there are 306 supercoiling-sensitive genes out of 4452 genes^14^). Meanwhile, genes responsible for long-term responses to cold-shocks (35 genes^42^) appear to be impervious to supercoiling (0% chances). The probability that these results would occur by random chance are 1.8% in the case of genes responsible for transient responses, and 8.5% in the case of genes responsible for long-term responses, as estimated from bootstrapped distributions (70 genes each) from 10000 random resamples with replacement using a non-parametric bootstrap method^66^. We thus conclude that both classes of cold shock-related genes have atypical sensitivity (even though opposite) to supercoiling.

## Discussion

Temperature-driven changes in genomic DNA supercoiling may be one of *E*. *coli*’s mechanisms for sensing and responding to temperature shifts^67^. A previous study^19^ using single-molecule mRNA FISH assays to show that, at optimal temperatures, DNA supercoiling buildup eventually halts transcription initiation, which can be resumed upon release of the supercoiling by Gyrase. This buildup only has significant effects in chromosomal genes (in highly expressing operons^61^), since plasmids lack discrete topological constraints, allowing the negative and positive supercoiling emerging in transcription to diffuse freely in opposite directions, until nullifying one another^19^.

It is expected that both the time needed for Gyrase to find DNA regions requiring intervention as well as the energy required for this process are temperature-dependent. As such, it may be that the effects of lowering temperature differ both between chromosome-integrated and plasmid-borne genes, as well as between chromosome-integrated genes with different transcription rates.

We studied this phenomenon in the P_LacO3O1_ promoter, under full induction, as a function of temperature (this promoter, due to lacking the O_2_ site and being under full induction, is not expected to form significant discrete topological constraints^30^). We showed that its response to temperature downshifts differs, depending on whether it is chromosomally-integrated or plasmid-borne. Specifically, the chromosome-integrated gene is more sensitive to critically low temperatures (below 23 °C), becoming weaker and noisier, and having comparatively longer-length steps preceding the open complex formation than the single-copy plasmid-borne gene. In particular, in these conditions, similar to when inhibiting Gyrase or Topoisomerase I, the transcription activity of the chromosomally-integrated gene is hampered, due to increased time to escape from positive supercoiling buildup. Overall, the results suggest that promoters’ in *E*. *coli* differ in sensitivity to shifts to critically low temperatures when chromosome-integrated and when plasmid-borne, due to the temperature-dependence of the kinetics of promoter locking due to positive supercoiling buildup. Simulations of a stochastic model of transcription with a temperature-dependent escape from the locked promoter state are consistent with these observations.

An indirect evidence for the existence of promoter blocking at low temperatures in the chromosome-integrated gene is the high cell-to-cell variability in integer-valued RNA numbers at 10 °C, when compared to the plasmid-borne gene (Table 1). This variability, one hour following induction, suggests that the kinetics of RNA production differed significantly between cells with the chromosome-integrated gene (in agreement with the occurrence of promoter locking in some cells and transcription activity in others). In particular, we estimate that cells with active promoters produced ~1 to 4 RNAs (consistent with mean RNA numbers of native active genes in *E*. *coli*^62^), while cells with locked promoters produced ~0 to 2 RNAs at most.

Importantly, we found evidence that the hampering of escape of P_LacO3O1_ from supercoiled buildup states at low temperatures is an energy-associated increase in difficulty to release locked promoters, rather than due to a reduced functionality of Gyrases or Topoisomerases I. First, inhibiting the activity of these proteins with Novobiocin and Topotecan at 10 °C further reduces transcription reactivation (agreeing with the suggestion that these proteins evolved to act in response to cold-shocks^37,68^). Also, cells with energy depletion due to DNP treatment exhibit a similar transcription dynamics to when at low temperatures. Finally, subjecting cells to consecutive shifts between high and low temperatures results in smooth transitions in the transcription dynamics, which would not be possible if the population of Gyrases or Topoisomerases I had to be renewed.

We do not know whether the transcriptional halting at low temperatures of the chromosome-integrated P_LacO3O1_ is enhanced by the known overexpression at low temperatures of H-NS and similar NAP proteins present in the nucleoid, which, in these conditions, appear to selectively inhibit early step(s) in transcription initiation by binding to the promoter and acting as transcriptional repressors^69^. However, the fast recovery of the kinetics of RNA production under the control of P_LacO3O1_ observed in Fig. 5, when changing temperature from 10 °C and 30 °C, could be an indication that these proteins are not involved in the phenomenon observed. Similarly, we also do not know whether there is any influence from stringent response mechanisms. Studies of the roles of, e.g., *dskA* and *ppGpp*, may prove to be of value to determine whether, e.g., the biophysical phenomena here reported are affected by these mechanisms.

It is well established that the influence of positive supercoiling buildup on a chromosome-integrated gene differs with its location in the chromosome, because this phenomenon is sensitive to the expression rates of the operons of a DNA loop^64^. The present study indirectly supports this, by showing that the sensitivity of a gene differs with its own transcription rate. In particular, when replacing the promoter (P_LacO3O1_) controlling the chromosome integrated gene by the native *lac*, of weaker activity, we observed much weaker effects when lowering temperature. Also, overexpressing Gyrase no longer had an effect, suggesting absence of positive supercoiling buildup. This can be explained by this gene’s longer time intervals between transcription events, which allow Gyrases to act so as to escape from positive supercoiling buildup^70^ prior to these having a significant impact. Thus, we hypothesize that the effects of temperature downshifts on positive supercoiling buildup are promoter activity dependent.

Since the effects of temperature downshifts differ with the kinetics of positive supercoiling buildup, which itself differs with the gene location in the chromosome^64^ and own activity level (among other variables), one can expect genome-wide heterogeneity in the response to temperature downshifts. Thus, it is possible that genes associated to the responses to cold-shocks have atypical sensitivity to positive supercoiling buildup. When investigating this possibility, we found that the number of genes that are responsible for transient responses to cold-shock and also have high-sensitivity to supercoiling buildup is above-expected. Further, the number of genes that are responsible for long-term responses that also have high-sensitivity is below-expected.

We interpret these results as follows. Long-term activity following a cold-shock should be facilitated if a gene is only weakly affected by supercoiling buildup (as observed here when comparing the kinetics of the chromosomally integrated P_LacO3O1_ and native *lac*). Meanwhile, above average sensitivity to supercoiling is expected to contribute to (or be responsible for) the *transient* nature of the response exhibited by some genes associated to cold-shock. In particular, for a gene to remain active for ~1 hour^42^ and then have its activity reduced, there is a need for a mechanism of slow repression, which would be consistent to the ‘repression’ caused by positive supercoiling buildup (similar to what is observed in Figs 2, 3 and 4, with activity shutdown occurring after 30–60 minutes).

Overall, our results suggest that it may be possible that the temperature-dependence of the kinetics of promoter locking due to positive supercoiling buildup could be used as a means to introduce temperature sensitivity in some transcriptional programs of *E*. *coli*. In this regard, this may be of value to chromosome-integrated, synthetic genetic circuits with temperature-sensitivity. For example, if locating component genes on different DNA loops (with different levels of transcriptional activity), it may be possible for temperature shifts to generate heterogeneity in the responses of the component genes, which can be used to trigger changes in the state of the circuit.

## Materials and Methods

*E*. *coli* strain BW25993 *(lacIq hsdR514 ΔaraBADAH33 ΔrhaBADLD78)*^71^ cells carry the target and reporter genes. The target gene is controlled by P_LacO3O1_ and codes for an array of 48 binding sites for a modified viral coat protein, MS2-GFP^72–74^. P_LacO3O1_, inducible by IPTG, was engineered from the *E*. *coli* native *lac* promoter, by removing the O_2_ repressor binding site downstream of the transcription start site^65^. Due to lacking the site O_2_, we expect little to no formation of significant topological constraints^30^. Also, the repression strength of *LacI* is expected to be 2–3 fold weaker than on the wild-type *lac* promoter^65^. Finally, in one measurement, we made use of a chromosome-integrated native *lac* promoter, also followed by an identical array of binding sites for MS2-GFP.

To compare the RNA production rate of P_LacO3O1_ when single-copy plasmid-borne and when chromosome-integrated, two strains were engineered from the original BW25993. One carries a single copy full F-plasmid (~11 kbp)^75^, pBELOBAC11 (target plasmid), unknown to form long-lasting bounds to the membrane and originally responsible for the expression of transient DNA-binding proteins^76,77^. In this, we inserted the target gene, P_LacO3O1_, coding for the bindings sites for MS2-GFP (Fig. S1 in Supplementary Information). In the other strain, the target gene, controlled by P_LacO3O1_, was integrated into the *lac* gene locus of *E*. *coli*’s genome using Red/ET recombination (performed by Gene Bridges, Heidelberg, Germany) (Fig. S2 and Tables S13 and S14 in Supplementary Information).

Both strains were also transformed with a medium copy reporter plasmid pZA25-GFP^78^ (kind gift from Orna Amster-Choder, Hebrew University of Jerusalem, Israel), coding for the reporter protein MS2-GFP controlled by the BAD promoter. The multiple MS2-GFP binding sites in the target RNAs and the strong binding affinity of each site allow target RNAs to appear as bright spots, soon after produced (Fig. S3)^74^. Their maximum fluorescence is reached in less than 1 min^61^ and, once reached, remains constant for hours^61^, due to lack of interference from RNA degradation^61,74^.

While the strain carrying the target gene in single-copy F-plasmid also contains a native *lac* promoter in the chromosome (and, thus, has higher number of *LacI* binding sites overall than the strain carrying the chromosome integrate target gene as the original *lac* promoter was replaced by the target one), both strains overexpress *LacI*, reducing the possibility of significant effects due to shortage of repressors in the strain carrying the F-plasmid. Further, our measurements were conducted under full induction (except the induction curves), further reducing any possibility of effects of differences in number of available repressors.

For overexpressing Gyrase, we engineered a plasmid (pZe11-P_rham_-gyrAB, with ampicillin resistance) with the *gyrA* and *gyrB* genes under the control of a Rhamnose promoter (Supplementary Section “Gyrase overexpression”).

Cell growth conditions, antibiotics, and means of induction of the target and report genes are described in Supplementary Information (section “Growth Conditions and Induction of the Reporter and Target Gene”).

When analyzing the data, we assumed that, in both strains, only 1 copy of the target gene is present in each cell. This approximation is based on our observation that, 1 hour after starting the measurements, only 15% and 12% of the cells had 2 nucleoids at, respectively, 10 °C and 30 °C (600 cells analyzed per condition) (Supplementary Information, section “Number of promoter copies during the cell lifetime”), suggesting that these cells with the chromosome integrated promoter only carry two copies of the target gene for a relatively short period of time during their lifetime. The same assumption is applied to the cells carrying the gene of interest in the single-copy F-plasmid. This is because F-plasmids replicate at the same time^79^ or shortly after^80^ the chromosome. Further, we measured the plasmid copy numbers by RT-qPCR relative to the number of chromosomes at any given time (Supplementary Information, section “Plasmid copy number calculation using RT-qPCR”). The measurements showed that the copy number of the single-copy F-plasmid (pBELO) relative to the chromosome copy number is 1.00 and 1.02 at 10 °C and 30 °C, respectively (Fig. S9 and Table S12), validating the assumption. Finally, we note that in neither strain did we find any significant difference in the rates of RNA production in the first and second half of the cells lifetime (which would be expected if the replication of the gene occurred early in the cell lifetime, e.g. at midpoint).

We quantified integer-valued RNA numbers in cells with repressed Gyrase activity and with repressed Topoisomerase I activity. For that, we used, respectively, Novobiocin and Topotecan^54,58,59^. Cells were grown as described in Supplementary Information. Following induction of the reporter gene, cells were incubated at the appropriate temperature (10 °C or 30 °C), at 250 rpm for 15 minutes, prior to induction of the target gene. Afterwards, 1000 μM of IPTG and 100 ng/μl of Novobiocin or 100 μM of Topotecan were added to the cells.

To determine RNA levels in cells treated with 2,4-Dinitrophenol (DNP) (which uncouples the oxidative phosphorylation, causing Adenosine triphosphate depletion)^81^ the growth and activation of reporter genes were carried out as described above. Next, 1000 μM of IPTG and 200 μM of DNP were added to the media and cells were incubated at 30 °C.

We used *E*. *coli* RL1314 strain to measure RNA polymerases (RNAP) intracellular concentrations, carrying RNAPs fused with GFP (RNAP-GFP)^82^. Changes in fluorescence levels (example image in Fig. S4A) with, e.g., media richness, are consistent with RT-PCR (rpoC transcript levels) and plate reading measurements^40^. To visualize the nucleoid we used 4′,6-diamidino-2-phenylindole (DAPI) (Fig. S4B)^70^.

We performed measurements in cells whose RNAP concentrations differ. To obtain such cells, we employed the method proposed in^40,43^. In short, we made use of M9 media differing in Glycerol concentration (Supplementary Information, section “Tuning intracellular RNAP concentrations”). Specifically, cells were grown in media with 0.2, 0.4, 0.6 and 0.8% of Glycerol, denoted as 0.5X, 1X (control), 1.5X and 2X, respectively. These conditions allow cells to differ in RNAP concentration while not differing in mean growth rates (assessed from the OD_600_ over time by a spectrophotometer), as visible in Fig. S8.

Images acquisition is described in Supplementary Information. It took, on average, ~3 minutes to move cells from the incubator to the microscope, assemble the imaging chamber with slides and cells, and start the observation. Images were then analyzed by the software iCellFusion^83^ and CellAging^60,84,85^ (example Figs S3 and S4).

Simulations of stochastic models of gene expression were performed by SGNS^86^, a simulator of chemical reaction systems whose dynamics is driven by the Stochastic Simulation Algorithm^87^ that allows multi-time-delayed reactions^88^.

## Supplementary information

Supplementary Material
