# Supplementary material for: Chromosome and plasmid-borne PLacO3O1 promoters differ in sensitivity to critically low temperatures
Source: Sci Rep. 2019 Mar 14;9:4486. doi: 10.1038/s41598-019-39618-z (PMC6418193; doi:10.1038/s41598-019-39618-z)
Supplement: Supplementary file 1 — Supplementary Material [file 41598_2019_39618_MOESM1_ESM.doc]

**Supplementary Material for: “Chromosome and plasmid-borne P_LacO3O1_ promoters differ in sensitivity to critically low temperatures”**

Samuel M.D. Oliveira^1^, Nadia S.M. Goncalves^1^, Vinodh K. Kandavalli^1^, Leonardo Martins^1,2^, Ramakanth Neeli-Venkata^1^, Jan Reyelt^3^, Jose M. Fonseca^2^, Jason Lloyd-Price^4,5^, Harald Kranz^3^, and Andre S. Ribeiro^1,2,+^

^1^ Laboratory of Biosystem Dynamics and Multi-Scaled Biodata Analysis and Modelling Research Community, Faculty of Medicine and Health Technology, Tampere University, Korkeakoulunkatu 7, 33720 Tampere, Finland..

^2^ CA3 CTS/UNINOVA. Faculdade de Ciências e Tecnologia, Universidade Nova de Lisboa, Quinta da Torre, 2829-516, Caparica, Portugal.

^3^ Gene Bridges, Im Neuenheimer Feld 584, 69120 Heidelberg, Germany.

^4^ Biostatistics Department, Harvard T.H. Chan School of Public Health, Boston, MA 02115, USA

^5^ Infectious Disease and Microbiome Program, Broad Institute, Cambridge, MA 02142, USA

^+^Corresponding author. Email: andre.sanchesribeiro@tuni.fi, Tel: +358408490736, Fax: +358331154989

**SI Materials and Methods**

***Growth Conditions and Induction of the Reporter and Target Gene***

From single colonies on LB agar plates, cells were cultured in LB medium with the appropriate concentration of antibiotics and incubated overnight at 30 °C and 250 rpm. These overnight cultures were then diluted to an initial optical density (OD_600_) of 0.05 in fresh M9 medium, with a culture volume of 20 ml supplemented with the appropriate antibiotics and 0.4 % of Glycerol (Sigma-Aldrich, USA), which was incubated at 37 °C with a 250 rpm agitation, until reaching an OD_600_ of ~ 0.3.

Regarding antibiotic controls, as we used the same reporter (plasmid-borne) in two strains differing in the location of the target gene, we also use the same antibiotic as a control (kanamycin). As a control for the target gene (when on the single-copy plasmid) we used chloramphenicol.

Next, to induce the expression of the reporter MS2-GFP proteins, 0.4% of L-Arabinose (Sigma-Aldrich, USA) was added and cells were incubated at 37 °C for 30 min with 250 rpm agitation. At this point, cells were incubated at the respective temperature (30 °C, 27 °C, 23 °C, 20 °C, 16 °C and 10 °C) (Innova® 40 incubator, New Brunswick Scientific, USA), for 15 min with agitation, to allow adaptation to a new temperature condition before activating the target gene. For all conditions, the temperature of the culture was constantly measured during the experiments using a thermometer, ensuring that the culture had reached and kept the desired temperature. Following full induction of the target gene (1000 μM IPTG, Sigma-Aldrich, USA), cells were incubated at the respective temperature for 1 hour, prior to image acquisition or RT-PCR measurements.

To obtain the induction curve of the target gene (Figure 1), cells were incubated with different concentrations of IPTG (0, 50, 100, 250, 500 and 1000 μM) for 1 hour, at 30 °C, before imaging.

To compare RNA production of the chromosome and plasmid genes at 10 °C and at 30 °C, cells were grown as previously described and incubated at 10 °C and 30 °C, respectively, for 15 minutes prior the induction of the target gene. Next, 1000 μM of ITPG was added. To determine the target RNA production level of both constructs, while keeping the temperature of the cultures constant, samples were taken right before adding the IPTG (time 0), and afterwards for every 15 minutes, for a total of 90 minutes.

We also quantified RNA numbers in cells with repressed Gyrase activity and with repressed Topoisomerase I activity. For that, we used, respectively, Novobiocin and Topotecan^1-3^. Cells with the target and reporter systems were grown as described below in the section ‘Growth Conditions and Induction of the Reporter and Target Gene’. Following induction of the reporter gene, cells were incubated at the appropriate temperature (10°C or 30°C), at 250 rpm for 15 minutes, prior to induction of the target gene. Afterward, 1000 μM of IPTG and 100 ng μl-1 of Novobiocin or 100 μM of Topotecan were added to the cells.

To determine the RNA levels in cells treated with 2,4-Dinitrophenol (DNP) (known to uncouple the oxidative phosphorylation, thus resulting in the depletion of Adenosine triphosphate)^4^ the growth and activation of the reporter genes were carried out as previously described. Next, 1000 μM of IPTG and 200 μM of DNP were added to the media and cells were incubated at 30°C.

***Tuning intracellular RNAP concentrations***

To measure intracellular concentrations of RNA polymerases (RNAP), we used *E. coli* RL1314 strain (a kind gift from Robert Landick, University of Wisconsin-Madison, USA), carrying GFP tagged RNAPs (RNAP-GFP)^5^.

To change intracellular RNAP concentrations in *E. coli* RL1314 cells, we followed the strategy proposed in^6^. Shortly, it consists of placing cells in media with differing richness that, for a limited range of media richness, results in different intracellular RNAP concentrations without significant differences in cell growth rates between conditions^6^.

To obtain cell populations with significantly different mean RNAP concentrations, instead of LB medium with various concentrations of tryptone and yeast extract as in^6^, we used M9 medium and supplemented it with different concentrations of Glycerol. We set M9 medium supplemented with 0.4% Glycerol as our ‘control’ medium. This condition is here denoted as ‘1X’. Overall, cells were grown in media with 0.2, 0.4, 0.6 and 0.8% of Glycerol, denoted as 0.5X, 1X, 1.5X and 2X, respectively.

For each of these media conditions, growth rates were assessed from the OD_600_ over time by a spectrophotometer. For cells with either construct, we found no significant differences in growth rates with changes in media (Figure S8).

Meanwhile, the observed changes in fluorescence levels (see example image in Figure S4A in Supplementary Material) with varying media richness are consistent with RT-PCR (*rpoC* transcript levels) and plate reading measurements^6^.

***Nucleoid staining with DAPI***

We assessed if the weaker transcriptional activity of the chromosome-integrated promoter at the lowest temperatures could be explained by changes in nucleoid. For this, starting from a single colony, cells were incubated overnight at 30 °C with agitation and aeration, in LB medium supplemented with 35 μg/ml Kanamycin. The overnight culture was then diluted to an initial OD_600_ of 0.05 in the respective M9 medium (0.5X, 1X, 1.5X and 2X), supplemented with Kanamycin, and grown at 37 °C until an OD_600_ of 0.3. At this point, cells were transferred and incubated at the appropriate temperature (10 °C, 16 °C, 23 °C and 30 °C), with agitation, for 75 minutes. Afterwards, cells were fixed with 3.7 % formaldehyde, at room temperature, and then centrifuged. To visualize the nucleoid, cells were resuspended in 1 X PBS, and 4’,6-Diamidino-2-Phenylindole (DAPI) (0.2 μg/ml) was added to this suspension. Next, cells were incubated at room temperature, for 20 minutes, and then washed twice with PBS. Cells were then resuspended in PBS, and 3 μl aliquots of these samples were placed on 2% agarose pads for imaging.

Results of these tests are reported in the main manuscript (section ‘Transcription by the chromosome-integrated construct is noisier at lower temperatures’)

***Gyrase overexpression***

For overexpressing Gyrase, we constructed a plasmid (pZe11 Prham-gyrAB-sfGFP, with ampicillin resistance) with the *gyrA* and *gyrB* genes under the control of a Rhamnose promoter. The genes were arranged in a polycistronic manner using their (identical) ribosome binding site to maintain the physiological stoichiometry of the two subunits. We amplified the *sfGFP* using the primers: Forward: 5’ CATATGAGCAAAGGAGAAGAACTTTT 3’, Reverse: 5’ CGGCCGTTTGTAGAGCTCATCCATGC 3’ with restriction enzymes, and cloned it after the *gyrAB* genes by digestion followed by ligation. The *sfGFP* was used to measure the amount of Rhamnose needed to fully express the Rhamnose promoter. For experiments, we removed the *sfGFP*, by single digestion with the restriction enzyme *PaeI*, followed by ligation. The final plasmid (pZe11 Prham-gyrAB, with ampicillin resistance) was used to overexpress Gyrase in the cells using 0.4% Rhamnose, which was found to fully express the Rhamnose promoter while not affecting the cells growth and morphology. Cells containing this construct were grown with 0.4% Rhamnose for 2 hours prior to imaging (sections “Growth Conditions and Induction of the Reporter and Target Gene” and “Microscopy”).

***RT-PCR***

Two sets of RT-PCR measurements were conducted. One to validate the microscope measurements at different temperatures for both target genes and the other to produce τ-plots. In both, the target gene is activated as described above and cells were grown as for the microscopy measurements. The reporter gene was not activated, as it was not necessary.

To determine the fold change of the target gene as a function of RNAP concentrations, the *E. coli* strain BW25993 was grown in 0.5X, 1X, 1.5X, and 2X media, as described in the section “Growth Conditions and Induction of the Reporter and Target Gene”. These measurements were conducted in cells kept at 10 °C and 30 °C (the lowest and highest temperature conditions in the microscopy measurements, respectively).

One hour after induction of the target gene, cells were fixed by adding the RNAprotect bacteria reagent (Qiagen, Germany), followed by enzymatic lysis with Tris-EDTA Lysozyme (15 mg ml^-1^) buffer (pH 8.3). From the lysates, the RNA content was isolated using the RNeasy purification kit (Qiagen) according to the manufacturer instructions. The RNA was then separated by electrophoresis, using 1% agarose gel stained with SYBR® Safe DNA Gel Stain (Thermo Scientific, USA). The RNA was found to be intact, with clear bands for the 16S and 23S ribosomal RNA. The RNA yield (~2 μg μl^-1^) and the A_260/_A_280_ nm ratio were determined by a Nanovue Plus Spectrophotometer (GE Healthcare Life Sciences, USA). The ratio obtained (2.0-2.1) is indicative of a highly purified RNA. To remove DNA contamination, the samples were treated with DNase I (Thermo Scientific, USA) following the manufacturer instructions. The cDNA was synthesized from 1 µg of RNA using the iScript Reverse Transcription Supermix (Biorad, USA) according to the manufacturer instructions.

cDNA samples (10 ng µl^-1^) were mixed with the qPCR master mix containing iQ SYBR Green Supermix (Biorad, USA) with primers (200 nM) for the target and reference genes. The 16S rRNA was used as a reference. Since the sequences of MS2-GFP binding sites consist of many repeats, for this we use the sequence in between the promoter sequence and these sites sequences (Supplementary Figure S2). Namely, the primers set for the target mRNA (mCherry) and reference (16S rRNA) genes were: mCherry (Forward: 5’ CACCTACAAGGCCAAGAAGC 3’, Reverse: 5’ TGGTGTAGTCCTCGTTGTGG 3’), 16S rRNA (Forward: 5’ CGTCAGCTCGTGTTGTGAA 3’, Reverse: 5’ GGACCGCTGGCAA CAAAG 3’).

The qPCR experiments were performed using a Biorad MiniOpticon Real-Time PCR System (Biorad, USA). The thermal cycling protocol used was 40 cycles of 95 °C for 10s, 52 °C for 30s, and 72 °C for 30s, with the fluorescence being read after each cycle. All reactions were performed in 3 replicates per condition. The PCR efficiencies of these reactions were greater than 95%. No-RT and no-template controls were used to crosscheck non-specific signals and contamination. The Cq values generated by the CFX Manager^TM^ Software were used to calculate the fold changes in the target gene, normalized to the reference gene, and its standard error using Livak’s 2^-ΔΔCT^ method^7^.

***Microscopy***

To image cells, cells with the target and reporter genes were grown as described above (section ‘Growth Conditions and Induction of the Reporter and Target Gene’). After, cells were pelleted and re-suspended in ~100 µl of the remaining media. Three microliters of cells were placed on a 2% agarose gel pad of M9 medium and kept in between the microscope slide and a coverslip. It took, on average, ~3 minutes to move cells from the incubator to the microscope and start the observation. This time includes the assembly of the microscope imaging-chamber containing the slides and cells.

Cells were visualized by a Nikon Eclipse (Ti-E, Nikon) inverted microscope with a 100x Apo TIRF (1.49 NA, oil) objective. Confocal images were taken by a C2+ (Nikon) confocal laser-scanning system, with a pinhole size set to 1.2 AU. For confocal images, the size of a pixel corresponds to 0.062 μm using a scan area resolution of 2048×2048 pixels. MS2-GFP-RNA “spots” and fluorescence from RNAP-GFP were visualized by a 488 nm laser (Melles-Griot) and an HQ514/30 emission filter (Nikon). Epiﬂuorescence images, for visualization of DAPI-stained nucleoids, were taken by a mercury lamp excitation and a DAPI ﬁlter cube (EX 340-380, DM 400, BA 435-485, Nikon). Phase contrast images were taken (for cells segmentation) by an external phase contrast system and DS-Fi2 CCD camera (Nikon). Size of the phase contrast images was 2560×1920 pixels, in which a pixel corresponds to 0.048 μm. Phase contrast and confocal images were taken once and simultaneously by Nis-Elements software (Nikon).

***Image Analysis***

Cell segmentation from the images was performed by the software “iCellFusion”^8^ (Supplementary Material, example Figures S3, and S4). iCellFusion first performs automatic cell segmentation from phase contrast images. The results were then manually improved. Next, iCellFusion conducts automatic inter-modal image alignment between the phase-contrast and the corresponding fluorescence images (see example Figure S7 in Supplementary Material).

Nucleoid (red channel) and the RNAP (green channel) intensity levels were obtained from the images by iCellFusion as well. For these, Principal Component Analysis (PCA)^9^ was used to normalize the major and minor axes lengths and the center coordinates of each cell, in order to plot the intensity distribution of the selected fluorescence levels along the major cell axis (by dividing each cell in normalized bins along the major axis). Example images of cells with fluorescent RNAPs and nucleoids are shown in Figures S7 (C) and S10 (D), respectively.

Nucleoid segmentation is made using the algorithm described in^10^, where a Gaussian approximation is applied. To detect the presence of one nucleoid or two separated nucleoids, we applied the Gradient Path Labelling algorithm^11^. This selection was manually inspected and corrected. After nucleoid detection and segmentation, principal component analysis was used to obtain the position, dimension and orientation of the nucleoid in each cell^10^.

Next, detection of RNA-MS2-GFP fluorescence ‘spots’ inside cells is performed automatically in each segmented cell by the software ‘‘CellAging’’^12^, using the Kernel Density Estimation (KDE) method (median filter).

To quantify the integer-valued RNA numbers in each cell from the total fluorescence intensity of the spots within, we used the method proposed in^13^. First, the cellular background fluorescence intensity is subtracted from the light intensity of each RNA-spot, accounting for the number of pixels occupied by the RNA-spot. To estimate the intensity of a single RNA-spot, from several measurements of RNA-spots intensities, a histogram of spots intensities is plotted and the intensity of the ﬁrst peak of the histogram is selected^13^. The integer-valued absolute number of RNAs in each spot is calculated by dividing the spot intensity with the intensity of the first peak of the histogram, followed by rounding to the nearest integer. This provides an integer-valued absolute number of RNAs in each cell^6,10,14-17^.

Examples of the results of cell segmentation and RNA-spots detection are shown in Figures S7 (A) and (B), respectively.

***Control tests of the RNA counting method***

First, in order to determine whether the smallest spots detected by microscopy and image analysis of the cells correspond to a single RNA, we assessed if cells not carrying the target gene while carrying the reporter produced any spots of similar intensity. As shown in Table S1, such ‘Fake spots’ were found to occur only very rarely and, importantly, in much lower numbers that in cells carrying a non-induced target gene, both in the case of chromosome integration and plasmid borne. This difference between the two latter conditions and the first condition shows that ‘leaky’ RNA production is the main responsible for ‘RNA spot detection’ in the two latter conditions, rather than being the appearance of ‘fake spots’ due to MS2-GFP ‘clumpiness’.

Second, one important of the method for RNA counting described in the previous section is that the smallest spots in the images correspond to a single RNA^13^. To strengthen this argument, we extracted the intensity of such spots from non-induced cells containing only one spot. We further merged all data from different measurements, at different induction levels, and again detected the smallest peak of fluorescence intensity from individual spots. This peak intensity was found to be indistinguishable (in a statistical sense) from the one detected using the other methodology.

Finally, it is important to notice that the RNA molecules with MS2-GFP binding sites, once tagged by MS2, do not degrade nor lose fluorescence intensity for the duration of our measurements^14,18^. We verified this by conducting new measurements (Table S2). We found that, during the measurement period, the fluorescence of tagged RNAs does not decrease significantly over time (gradually or abruptly), in any construct or temperature, in agreement with previous reports^14,18^. Also, tagged RNAs from the chromosome integrated gene and the plasmid borne gene have nearly indistinguishable lifetime times, which only differ (mildly) with temperature (Supplementary Information, section “Half-life of the fluorescence intensity of MS2-GFP tagged RNA molecules”). In addition, we verified if the time for tagged RNAs to become fully tagged differed between strains or temperature conditions. In all cases, the total fluorescence of individual tagged RNAs was found to be already maximal when first detected, meaning that the full tagging takes always less than 1 minute (measurements taken once per minute), as in previous measurements^18^.

***Model of transcription kinetics***

First, we consider chromosome-integrated promoters. We assume the model of transcription whose rates have been empirically validated in ^6^ and ^19^. The model includes active transcription, a repression mechanism, and accounts for the effects of DNA super-coiling.

Active transcription^20,21^ consists of a multi-step process (reaction 1) that starts with the binding of an RNA polymerase (R) to a free, active promoter (P_ON_). This results in the formation of an unstable closed complex, RP_c_^22^. The reversibility of this step allows multiple occurrences of the closed complex formation between the occurrences of two transcription events.

Once a closed complex successfully leads to the initiation of an open complex (RP_o_) formation, the process becomes nearly irreversible^23^. Once the DNA double helix is opened and the open complex is formed, the RNA polymerase moves to elongation (clearing the promoter for additional initiation events), and finally, it completes RNA production^13,14,19,22-29^. Promoter clearing may be preceded by the production of a few short RNA transcripts (<10 nt) from abortive initiation events^26,30^, but only in rare promoters, with short-living open complexes, are these events rate limiting^31^. As such, these events are not represented here.

Note that the steps in reaction 1 do not represent elementary transitions, but rather the effective rates of rate-limiting steps^6^. E.g., k_1_ is the rate at which R binds to the promoter, but its value includes the influence of the time spent in non-speciﬁc bindings to the DNA and 1D diffusive searches^32^. We refer to this collection of events as the closed complex formation. Meanwhile, k_2_ is the rate at which a series of steps is completed once initiated^26^. As mentioned above and in accordance with the literature^25^, we refer to these steps as the open complex formation. Finally, note that, in reaction 1, when the forward rate of a given step is much faster than its backward rate, the negligible reversibility is not represented:

(1)

Aside from the process of active transcription, there is a repression mechanism, due to the action of LacI molecules^6,23^, accounted for by reaction 2:

(2)

Note that we expect that, when the promoter is under full induction (which is the condition that we focus on in the present study), the effects of repression will be very mild in the overall process of RNA production.

Finally, in reaction 1, following a transcription event, it is assumed that the promoter changes directly into a ‘locked’ state, making it unable to initiate a new closed complex until released by the action of Gyrase or Topoisomerase I, depending on the nature of the supercoiled state^13,19^. This model of locking by DNA super-coiling is, to an extent, ‘simplistic’, since this is a cumulative process that does not necessarily occur after each transcription event (as the model assumes), only occurring after “one to a few” events^19^ (i.e. it is a stochastic, cumulative process). However, as shown in the results section, this approximation does not affect the ability of the model to match the empirical data for the entire range of conditions tested, suggesting that it takes very few events for locking to occur^19^ (as the results reported in ^19^ suggest).

The process of ‘unlocking’ from super-coiling states is modeled by reaction 3 (Gyrase and Topoisomerase I are not explicitly represented due to their large copy numbers^19^):

(3)

The rate of this reaction, k_unlock_, is assumed the most temperature-dependent variable of this system of three reactions.

Meanwhile, in the case of plasmid-borne promoters, we assume a similar model but with no super-coiling effects, in agreement with^19^. The model of transcription of plasmid-integrated promoters can thus be obtained from the above by allowing the promoter to change to a P_ON_ state following a transcription event, or equivalently by setting k_unlock_ to ‘infinite’.

In normal conditions, the subsequent steps of elongation, termination and RNA release^20,33-35^, are much faster than initiation^15,36-40^ in both plasmids and in the chromosome. Thus, k_3_ is assumed ‘infinitely’ fast. Further, regardless of the duration of these steps; note that rate-limiting steps in elongation should not affect the mean of the time-length of the intervals between consecutive RNA productions, only their variance^41^.

Finally, simulations of the stochastic models of gene expression were performed by SGNS^42^, a simulator of chemical reaction systems whose dynamics is driven by the Stochastic Simulation Algorithm^43^ but that allows for multi-time-delayed reactions^44^. SGNS also allows hierarchical, interlinked compartments to be created, destroyed and divided at runtime, a feature used to generate dynamically independent model cells that can differ in any of the model features, such as an initial number of molecular components, to lifetimes, to rate-constant values, etc.

***Lineweaver-Burk Plots***

In short, this method^6,16^ is similar to steady-state assays designed to investigate the *in vitro* dynamics of transcription in bacteria^22^. The methods are based on the assumption that increasing the concentration of active RNAP molecules in the cell (or vessel of reactions), one should increase the rate of RNA production. This increase in RNA production rate is known to occur due to the increased rate of the steps prior to the start of the open complex formation (which depend on RNAP numbers, see reaction 1 in this Supplementary Material), while the rate of the steps following the start of open complex formation is not altered. Given this, one can assume that, for infinite RNAP numbers, the rate-limiting step of transcription are solely the steps after the open complex formation.

To obtain a Lineweaver-Burk plot, we plot the inverse of the RNA production rate against the inverse of the RNAp concentrations, for various conditions differing in the concentration of RNAP in the cells (see section “Growth Conditions”). Next, we fitted a line to the points from which we obtain the relative mean duration prior to and following commitment to transcription as described in the next section.

In addition to this, we tested whether the cells in different media richness conditions differed in other morphological or physiological parameters.

First, to show that the differences in the media conditions do not generate differences in the cells’ DNA spatial organization, we obtained the DAPI signal in each cell for each of the 0.5X, 1X, 1.5X and 2X conditions, for cells at 30 °C and 10 °C. Then we tested (KS-tests), whether the single-cell distributions of these fluorescence levels differed between conditions. Results in Table S6 show that the distributions cannot be distinguished, in a statistical sense (except when comparing, at 10 °C, the 0.5X and the 2.0X conditions directly).

Second, rather than the RNAP intracellular concentration, it is the relative free RNAp concentration that determines the transcription rate. Thus, we measured RNAp concentrations from the RNAP-GFP signal intensity. Next, we determined whether, in the conditions studied here, the relative differences in RNAp-GFP concentration between conditions (Supplementary Table S7) are a good proxy for the relative differences in free RNAp concentrations and, thus, can be used to assess the ability of RNAP to synthesize mRNA in the cells. For this, we tested if, for the range of media richness used, the inverse of the RNA production rates (Supplementary Table S9) change linearly with the inverse of the RNAP concentrations. I.e., a Lineweaver–Burk plot^45^ should exhibit a line, as this indicates that the relative free RNAp concentrations can be approximately assessed from the total RNAp concentrations, and that no factors other than the changes in the free RNAp concentration affect the promoter of interest. This also indicates that, unlike other cases^46^, in our measurements the RNAP foci (here RNAP-GFP) is adequate in reflecting the ability of RNAp to synthesize mRNA.

Third, once the Lineweaver-Burk plot is obtained, it is important that the linear relationship between the inverse of the RNAP concentration and the inverse of the RNA production rate is tested. Here, we test this by a likelihood ratio test, to determine whether the small deviations from linearity are statistically signiﬁcant (we used a weighted total least-squares algorithm for ﬁtting a straight line, WTLS^47^). From Figure S10, the linear relationship can be observed for the both chromosome and plasmid constructs. In agreement, the results of the linearity tests show that, within this range of RNAp concentrations, the linear model cannot be rejected in either case (P > 0.25).

Given all of the above, we conclude that, in the measurements presented here, the relative free RNAp concentrations in the cells are well-approximated by the total RNAp concentrations, and that there are no other factors affecting signiﬁcantly the initiation dynamics of the P_LacO3O1_ promoter.

***Relative mean duration prior to and following commitment to transcription***

We use a recently developed technique to dissect the *in vivo* kinetics of transcription initiation in live *E. coli* cells^6,16^, based on previous *in vitro* techniques^14,22,25^. This method allows us to estimate the mean fraction of time between consecutive transcription events that is taken by the steps *following* the commitment to the open complex formation.

Given our model of transcription, let t_prior_ be the mean time for a *successful* closed complex formation, i.e. it is the mean time-length of all events prior to the commitment of the RNAP to open complex formation. As such, t_prior_ includes the expected time in OFF state as well as the time taken by multiple (failed) attempts to form a stable closed complex. Note that the kinetics of these steps depends on the RNAP intracellular concentrations.

Meanwhile, the remaining time to produce an RNA is denoted t_after._, and includes all steps *following* the commitment to the open complex formation, e.g. isomerization^21^, and prior to commitment to transcription elongation. Relevantly, the kinetics of these steps does not depend on the RNAP intracellular concentrations. Given this, the mean time interval between consecutive RNA productions (Δt) is:

$\Delta t=t_{prior}+t_{after}$ (4)

Since only t_prior_ is affected by a change in RNAP concentration, the new mean interval between consecutive RNA productions after such a change can be written:

$\Delta t^{new}=t_{prior}^{new}+t_{after}$ (5)

Where:

(6)

with

$S=\frac{\left[RNAP\right]^{new}}{\left[RNAP\right]}$ (7)

From this, one can write:

$\frac{\Delta t^{new}}{\Delta t}=\frac{S^{-1}\times t_{prior}+t_{after}}{t_{prior}+t_{after}}$ (8)

Next, assume that in the new condition the cells contain an infinite number of RNAPs (same assumption as in^6^). Given this, S^-1^ becomes null and, from equation 8:

(9)

Given this, from the normalized mean interval between RNA productions and the inverse of the relative RNAP concentration for a few conditions differing in RNAP concentrations (section ‘Tuning intracellular RNAP concentrations’ in Experimental Procedures), one can extrapolate the ratio , thus obtaining . This extrapolation, done here with the method of Weighted Total Least Squares^47^ with error in both coordinates, is valid if *Δt* changes linearly with changes in the inverse of the RNAP concentrations (shown to be valid within a certain range of media richness^6,16^). From this, one can also obtain the fraction of time prior to commitment to the open complex formation:

(10)

Here, the empirical values of Δt are estimated from the inverse of the RNA production rates obtained by RT-PCR, as in^16^. While these values are relative to a reference gene (here 16S RNA), the ratio between rates (*r*) in the ‘new’ and ‘control’ conditions equal the inverse of the ratio between the time intervals between consecutive RNA productions in individual cells, since RNA degradation rates do not differ with media richness within the range employed here^48^.

***Confidence Intervals***

Using Fieller's theorem^49^, we can derive the 90% CI of the ratio between the reference condition B and the tested condition A^50^ as follows:

$90\%CI=\left(\frac{mean(A)}{mean(B)}\right)\pm\left(t*\times\left(\left(\frac{mean(A)}{mean(B)}\right)\times\sqrt{\left(\frac{\left(\widehat{\sigma}_{A}\right)^{2}}{mean(A)^{2}}\right)+\left(\frac{\left(\widehat{\sigma}_{B}\right)^{2}}{mean(B)^{2}}\right)-2\left(\frac{\left(\widehat{\sigma}_{A,B}\right)}{mean(A)*mean(B)}\right)}\right)\right)$ (11)

Where *t^*^* is the critical Student's t-value for the degrees of freedom of the sum of A and B samples minus 2. Since we perform two-side tests, we search the table for the critical values for a probability of 0.95.

***Number of promoter copies during the cell lifetime***

In general, we assume that there is only one copy of the target promoter in a cell at all times, for both strains. To determine the extent to which this assumption is accurate, we measured the fraction of cells containing two chromosomes, 1 hour after the start of the microscopy measurements. Since, in *E. coli*, single-copy F-plasmids replicate at the same time^51^ or shortly after^52^ the chromosome, we only measured chromosome numbers, both in cells carrying the gene of interest in the chromosome as well as in cells carrying the gene of interest in the single copy F-plasmid.

For this, cells of both strains were grown as described in Experimental Procedures. Next, they were fixed and stained with DAPI to assess the location and size of nucleoids in live cells (Experimental Procedures, section “Tuning intracellular RNAP concentrations and nucleoid staining with DAPI”).

From images taken 1 hour after the start of the microscopy measurements, cells and nucleoids were segmented as described in Experimental Procedures (section ‘Image Analysis’). Results are reported in the third paragraph of the Results section. An example image of DAPI-stained nucleoid(s) is shown in Figure S7-D in Supplementary Material.

***Plasmid copy number calculation using RT-qPCR***

To assess the plasmid copy number, we followed the method described in ^53^. Briefly, it allows the absolute quantification of plasmid copy number using a standard curve that correlates the copy number of a gene with the C_T_ (cycle threshold) value obtained from RT-qPCR measurements. For this, the separate detection of the plasmid and the host strain chromosomal DNA is required, which is achieved by using two primer sets, one specific for a single copy gene present in the plasmid and the other present in the host strain chromosome.

Here, we used two sets of primers, one for the Chloramphenicol acetyltransferase (*cat*) gene (a single copy gene on the pBELO plasmid), and another for the *lacI* gene (a single copy gene on the *E. coli* BW25993 chromosome). For obtaining the curve, these genes were inserted in the same plasmid (gene ratio 1:1), which then can be used to calculate the copy number of the plasmid of interest. Since *cat* and *lacI* are single-copy genes, the plasmid copy number is the ratio of *cat* to *lacI*.

Cells carrying the plasmid-borne gene were grown as described in the section “Growth Conditions and Induction of the Reporter and Target Gene” of the Experimental Procedures. After the induction of the target gene (at 10°C and at 30°C), the total DNA content of the cells was extracted using the QIAamp DNA Mini kit (Qiagen) following the method for bacterial cells as per manufacturer’s instructions.

For the construction of the standard curves, we used the plasmid pCA24N-ligase^54.55^, which carries the coding sequence for both the *cat* and *lacI* genes. This plasmid was purified from an overnight culture grown in LB medium at 37°C, using the QIAprep Spin Miniprep kit (Qiagen).

The DNA concentration of both samples (the plasmid and the extracted DNA from *E. coli* BW25993 cells) was determined using the Qubit 4 Fluorimeter (ThermoFisher Scientific) and the Qubit 1X dsDNA HS kit (ThermoFisher Scientific).

A 10-fold serial dilution of the pCA24N-ligase plasmid, ranging from 10^4^ to 10^9^ copies μl^-1^, was performed to obtain the standard curves for the *cat* and *lacI* genes. After determining the plasmid concentration in each of these dilutions, the plasmid copy number was calculated from^53,56^:

$\text{DNA(copy)}=\frac{6.02\times10^{23}\text{(copy/mol)}\times\text{DNA amount (g)}}{\text{DNA length (dp)}\times660\text{(g/mol/dp)}}$ (12)

The real-time qPCR was performed using a Biorad MiniOpticon Real-Time PCR System (Biorad, USA). The RT-qPCR reaction mixture contained the iQ SYBR Green Supermix (Biorad, USA), the DNA template, and the primers for the target (*cat*) and reference (*lacI*) genes at a final concentration of 200 nM. The primers for the *cat* gene were (Forward: 5’ ATTCACATTCTTGCCCGCC 3’ and Reverse: 5’ CACCGTAACACGCCACATC 3’) and for the *lacI* were (Forward: 5’ ACCAGGATGCCATTGCTGTG 3’ and Reverse: 5’ TTTATGCCAGCCAGCCAGAC 3’), with the amplicon size being 209 and 221, respectively. The thermal cycling protocol used was: denaturation at 95°C for 3 min, followed by 40 cycles of 95°C for 10s, 58°C for 30s and 72°C for 30s, with the fluorescence being read at the end of each cycle. For all the samples tested, (the DNA extracted from *E. coli* BW25993 cells and for each of the pCA24N-ligase serial dilution), the reaction was conducted in triplicates, with a final volume of 25 μl. No-template controls were used to crosscheck for non-specific signals and contamination. After the amplification, a melting curve analysis, with a temperature gradient of 0.5°C/s from 65°C to 95°C, was performed to confirm the specificity of the amplification. The C_T_ values generated by the CFX Manager^TM^ Software were then used to obtain the standard curve for both genes, where these C_T_ values were plotted against the logarithm of their initial template copy numbers, determined using equation (12). In addition, the C_T_ obtained from the DNA extracted from *E. coli* BW25993 cells was used, along with the standard curve to determine the pBELO plasmid copy-number.

Each standard curve was generated using a linear regression of the plotted data. From the slope of each standard curve, the amplification efficiency (E) was determined from ^53^:

$E=10^{-1/\text{slope}}-1$ (13)

The standard curves for *cat* and *lacI*, ranging from 10^4^ to 10^9^ copies μl^-1^, are presented in Figure S9 in Supplementary Material. Both curves were linear, in the tested range, with a R^2^ > 0.997 and 0.999, respectively. The slopes of each standard curve were -3.12 and -3.08, for *cat* and *lacI*, respectively. From these, we determined the amplification efficiency for both genes, with the results being 1.10 for both *cat* and *lacI*.

The absolute quantification of the plasmid copy number was determined using the standard curves in Figure S9. The copy number of the *cat* and *lacI* genes in the total DNA extracted from *E. coli* BW25993 cells was determined from the corresponding standard curve, using the C_T_ values. The plasmid copy number of the pBELO was then calculated by dividing the copy number of *cat* by the copy number of *lacI*, given that both are single copy genes of pBELO and BW25993 chromosome, respectively. Thus, the ratio of *cat* to *lacI* is equal to the plasmid copy number of pBELO. The results from this quantification are shown in Table S12 and described in the Results section.

***Half-life of the fluorescence intensity of MS2-GFP tagged RNA molecules***

We observed several cells with a single MS2-GFP tagged RNA molecule for 1 hour, since the moment the tagged RNA was first detected. Next, we fitted the intensity of each such RNA over time with a decaying exponential function and inferred the degradation rate of its fluorescence intensity. We obtained the decaying rates, calculated the mean of these rates and then converted it into the mean half-life of MS2-GFP tagged RNAs.

The results in Table S2 in Supplementary Material agree with previous analyses of the coat protein of bacteriophage, MS2, which showed that the MS2 binding sites on the RNA are constantly occupied by MS2-GFP proteins provided that these exist in sufficient abundance, resulting in the ‘immortalization’ of the target RNA due to isolation from RNA-degrading enzymes for time-series microscopy measurements of 1-2 hours (see e.g.^13^).

**Supplementary Figures**


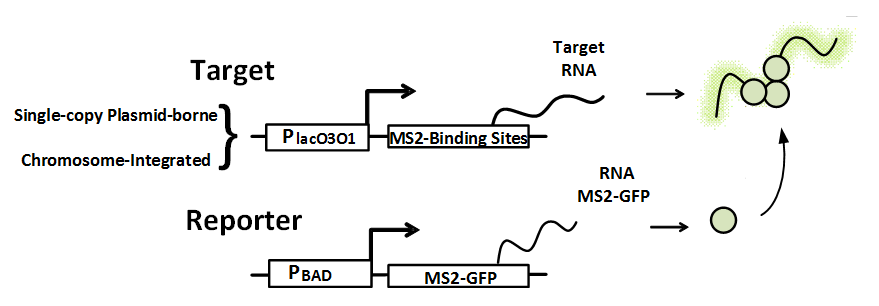


**Figure S1.** Single-RNA detection system schematic. Single-RNA detection system using MS2-GFP reporter proteins, whose production is controlled by P_BAD_, when applied to an RNA coding for multiple MS2 binding sites (BS), whose production is controlled by P_LacO3O1_ integrated into the chromosome, and when applied to an RNA with multiple BS for MS2-GFP whose production is controlled by P_LacO3O1_ integrated into a single-copy plasmid. In both systems, when individual target RNA molecules are produced, they are rapidly tagged by multiple MS2-GFP proteins produced by the reporter plasmid, making each RNA target for MS2-GFP visible under the fluorescence microscope as a fluorescent ‘spot’.

**
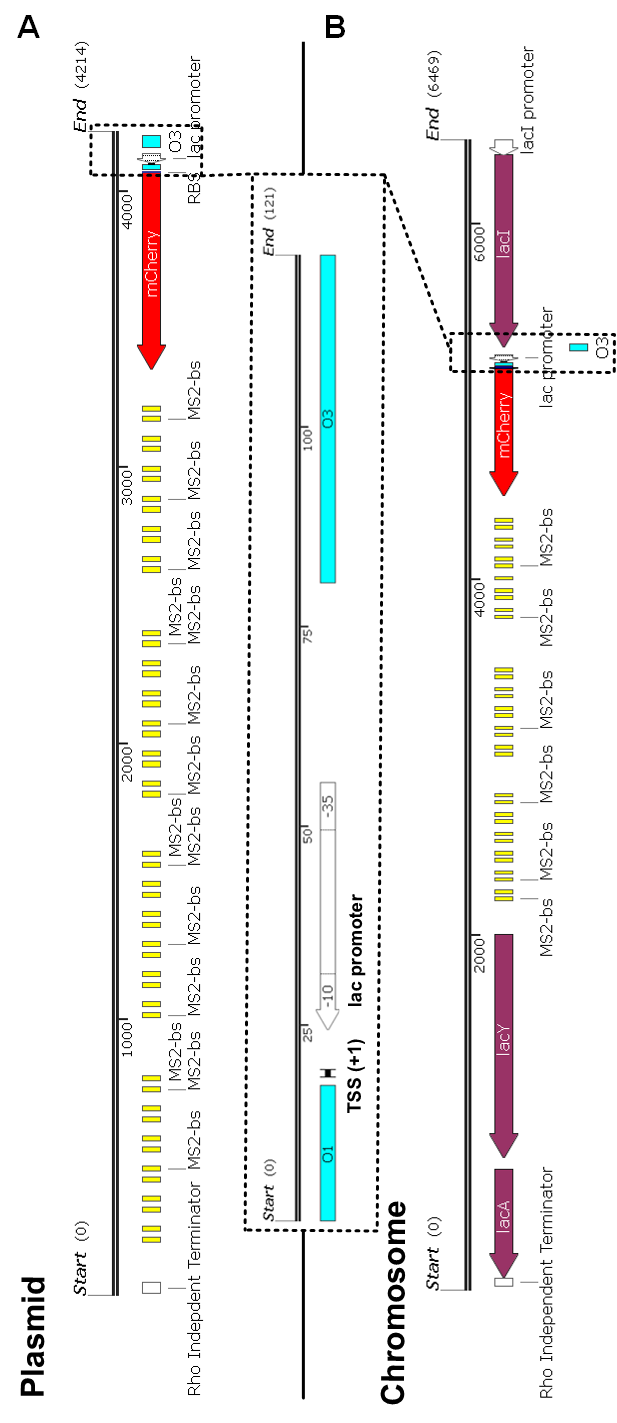
**

**Figure S2.** Genetic constructs. (A) Single-copy plasmid-borne gene (expected length of 4019 base pairs). MS2-BS cassette (depicted in yellow) under the control of P_LacO3O1_ promoter in a single-copy F-Plasmid in *E. coli* strain BW25993, followed by a Rho-Independent transcription termination site. (B) Chromosome-integrated gene (expected length of 5116 base pairs). MS2-BS cassette (depicted in yellow) under the control of P_LacO3O1_ promoter in the *lac* gene locus of *E. coli* strain BW25993 (ΔlacZ:MS2-BS), followed by the native *lacY* and *lacA* genes (depicted in purple), and the native Rho-Independent transcription termination site (depicted in white boxes). Constructs were confirmed by sequencing. Expected lengths are obtained from the difference between the position of the RBS (Ribosome Binding Site) and the downstream terminator sequences of the gene being transcribed. As the plasmid carrying the target gene does not code for *lacY* and *lacA*, and the cells carrying this plasmid also contain the original *lacY* and *lacA* genes in the chromosome, the two strains express *LacY* and *LacA* proteins similarly, and thus do not differ significantly in the dynamics of intake of IPTG. (Inset) The inset image in between the images of the two constructs shows in detail the P_LacO3O1_ promoter with functional domains, which is identical in both A and B constructs. It is in this identical region for both constructs that is located the operator site O3 (operator sites depicted in blue), followed by the *lac* promoter’s RNAp binding regions (starting from positions -10 and -35), the transcription start site (TSS, at +1 position), and the operator site O1. Note the mCherry sequence in between the promoter and the sequence coding for the MS2-BS in both constructs (depicted in red). Finally, note that in the plasmid construct there is a terminator upstream of the TSS, 27 nucleotides long, located 9 nucleotides downstream of the CmR gene (not represented in the figure), so as to be similar to the chromosome-integrated construct, where there is an upstream transcriptional terminator provided by the *lacI* gene.


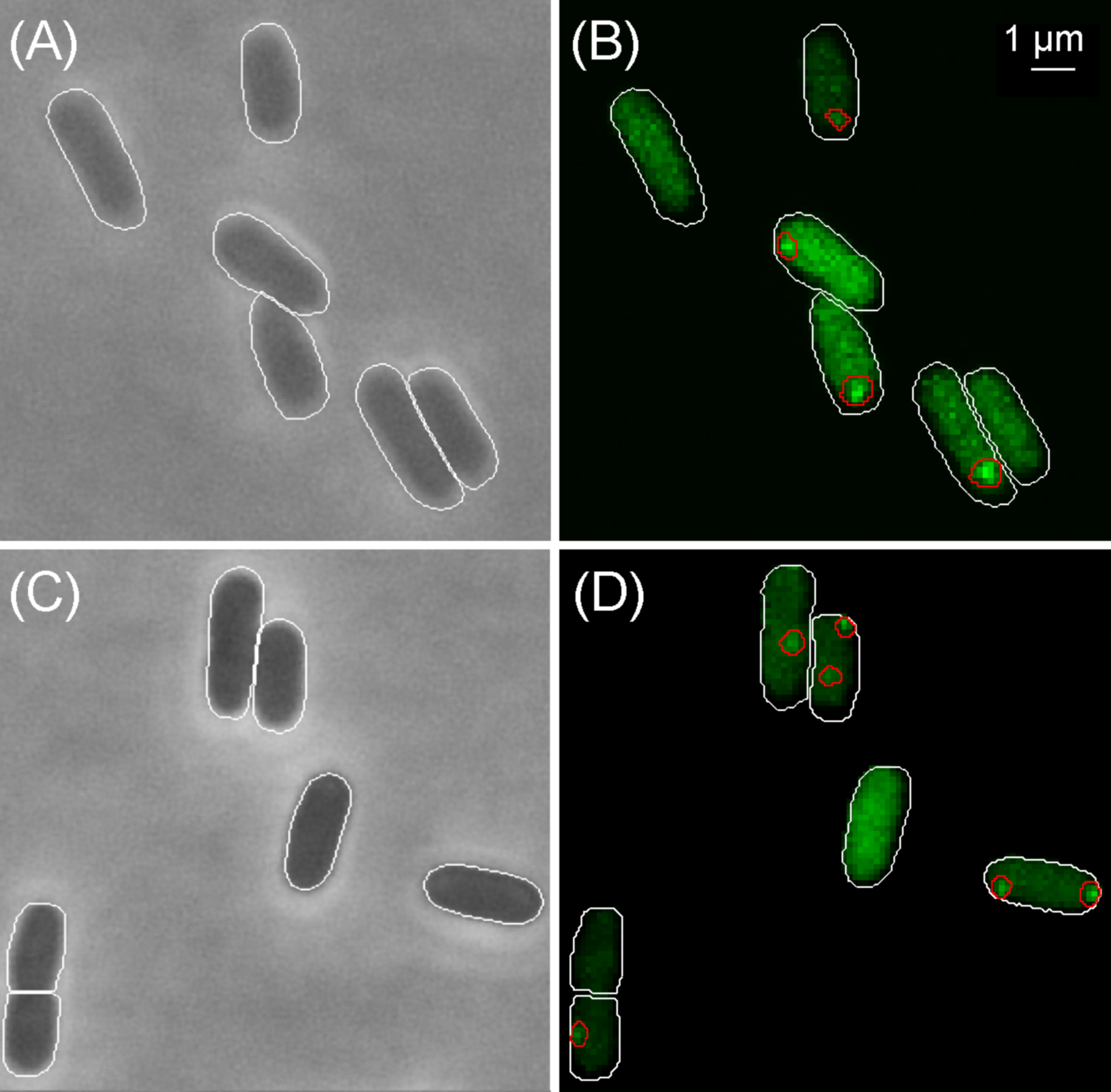


**Figure S3.** Example Microscopy Images. (A) Phase contrast image of cells at 30 °C along with results of semi-automatic cell segmentation. (B) Confocal microscopy image of the same cells at 30 °C with MS2-GFP tagged RNA spots, detected and segmented by the automatic spot detection method. (C) Phase contrast image of cells at 10 °C along with results of semi-automatic cell segmentation. (D) Confocal microscopy image of the same cells at 10 °C with MS2-GFP tagged RNA spots, detected and segmented by the automatic spot detection method. Note the scale bar in (B), which applies to all figures.


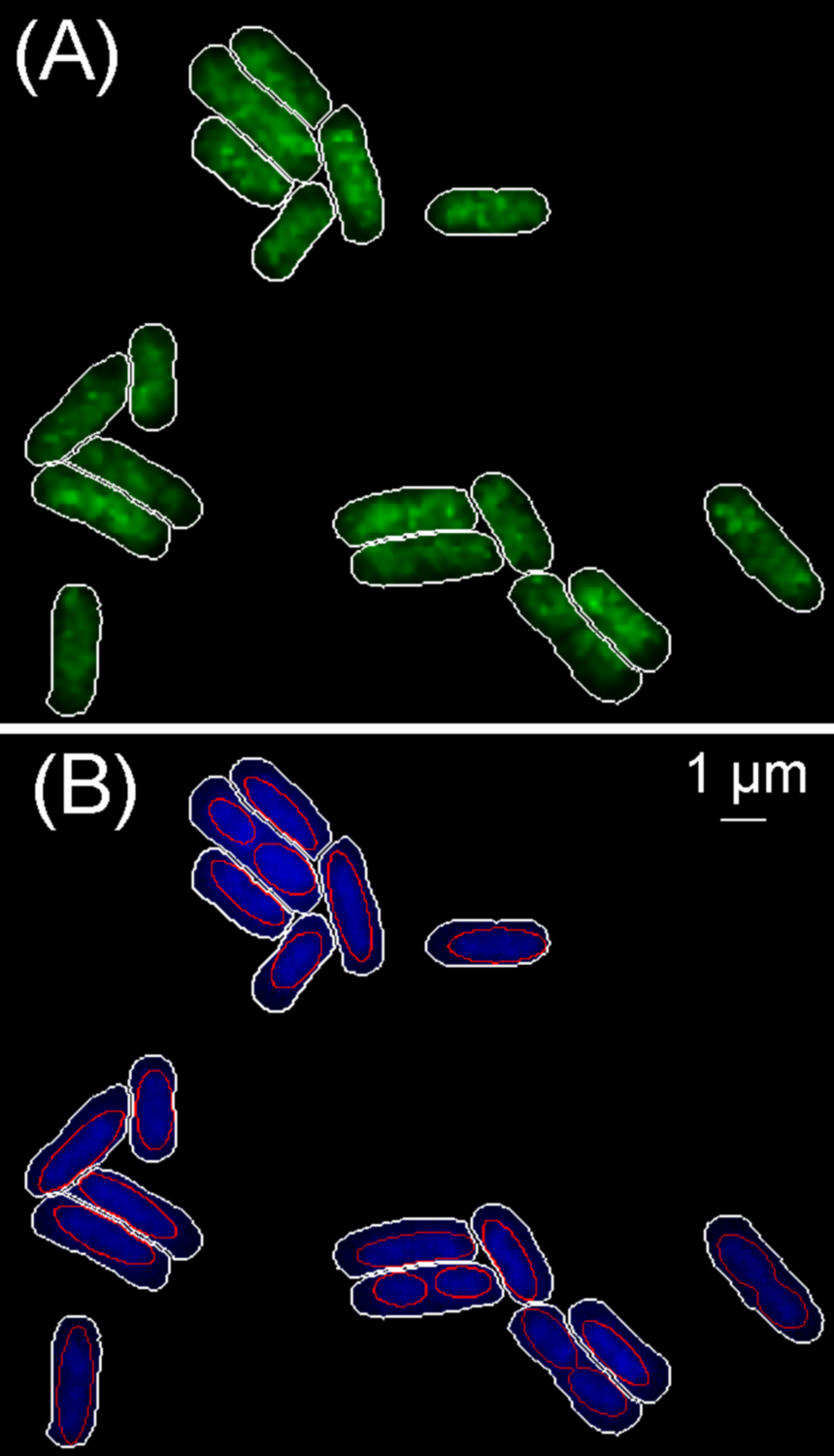


**Figure S4.** (A) Confocal microscopy image of cells with GFP-tagged RNA Polymerases. (B) Epifluorescence microscopy image of the same cells with DAPI-stained nucleoids. Note the scale bar in (B), which applies to both figures.

**
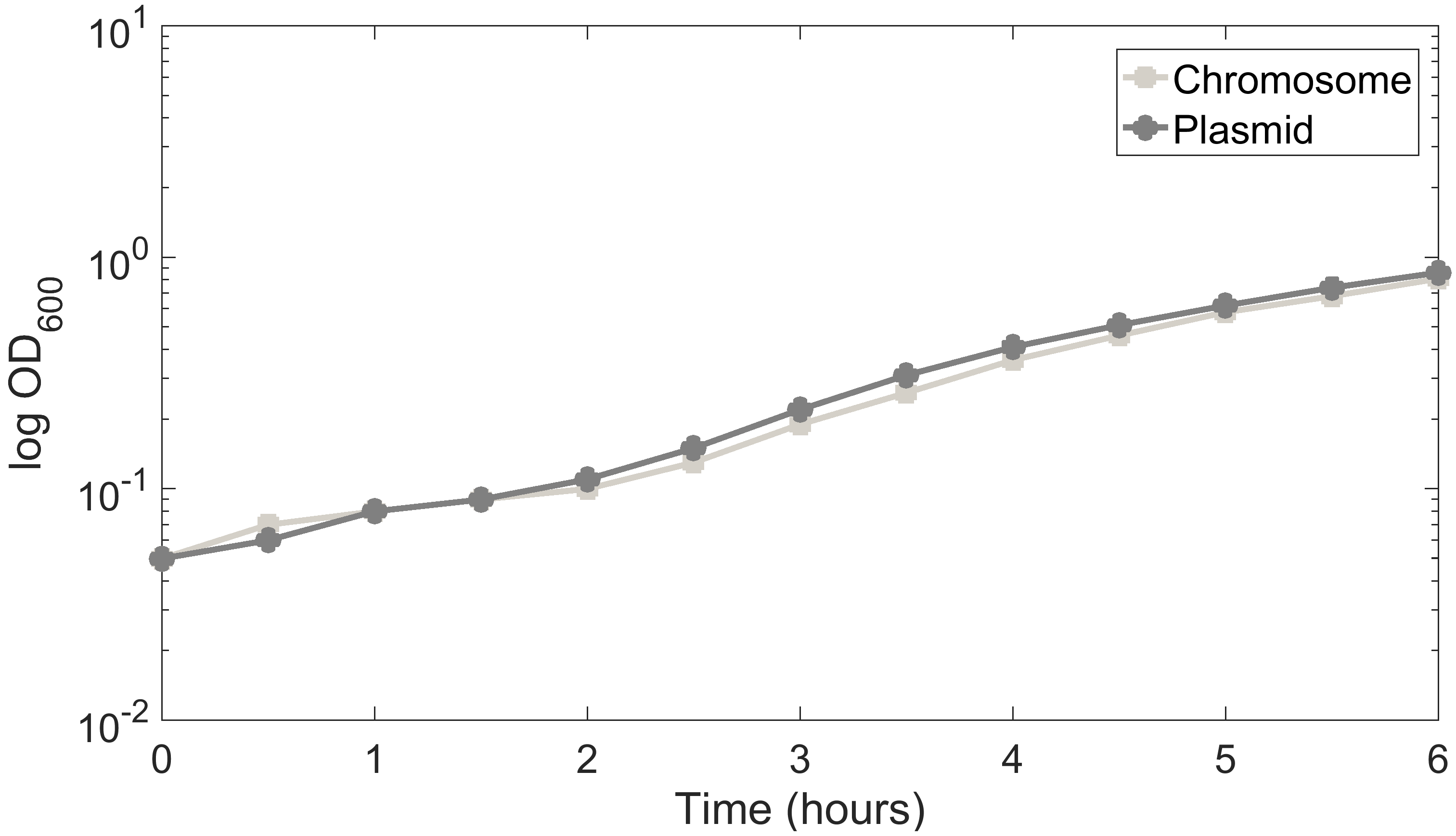
**

**Figure** **S5.** OD curves of cell populations in fresh media at 30 °C. The original *E. coli* strain where both the ‘Plasmid’ and ‘Chromosome’ constructs were inserted is BW25993. From a -80 °C glycerol stock, cells with the target and reporter genes were placed in LB medium agar plates with 34 μg/ml Chloramphenicol and 35 μg/ml Kanamycin (Sigma-Aldrich, USA) and incubated overnight at 37 °C. From these plates, a single colony was picked and cultured overnight at 30 °C, with agitation (250 rpm) and aeration, in LB medium supplemented with the appropriate concentration of antibiotics. From the overnight culture, cells were diluted to an initial optical density (OD_600_) of 0.05, in fresh M9 medium supplemented with the appropriate antibiotics and 0.4 % of Glycerol (Sigma-Aldrich, USA), and were incubated at 37 °C until reaching an OD_600_ of 0.3 (for the first 3 hours). They were then placed at 30 °C for 3 hours. The OD_600_ was then measured every 30 minutes for 6 hours.


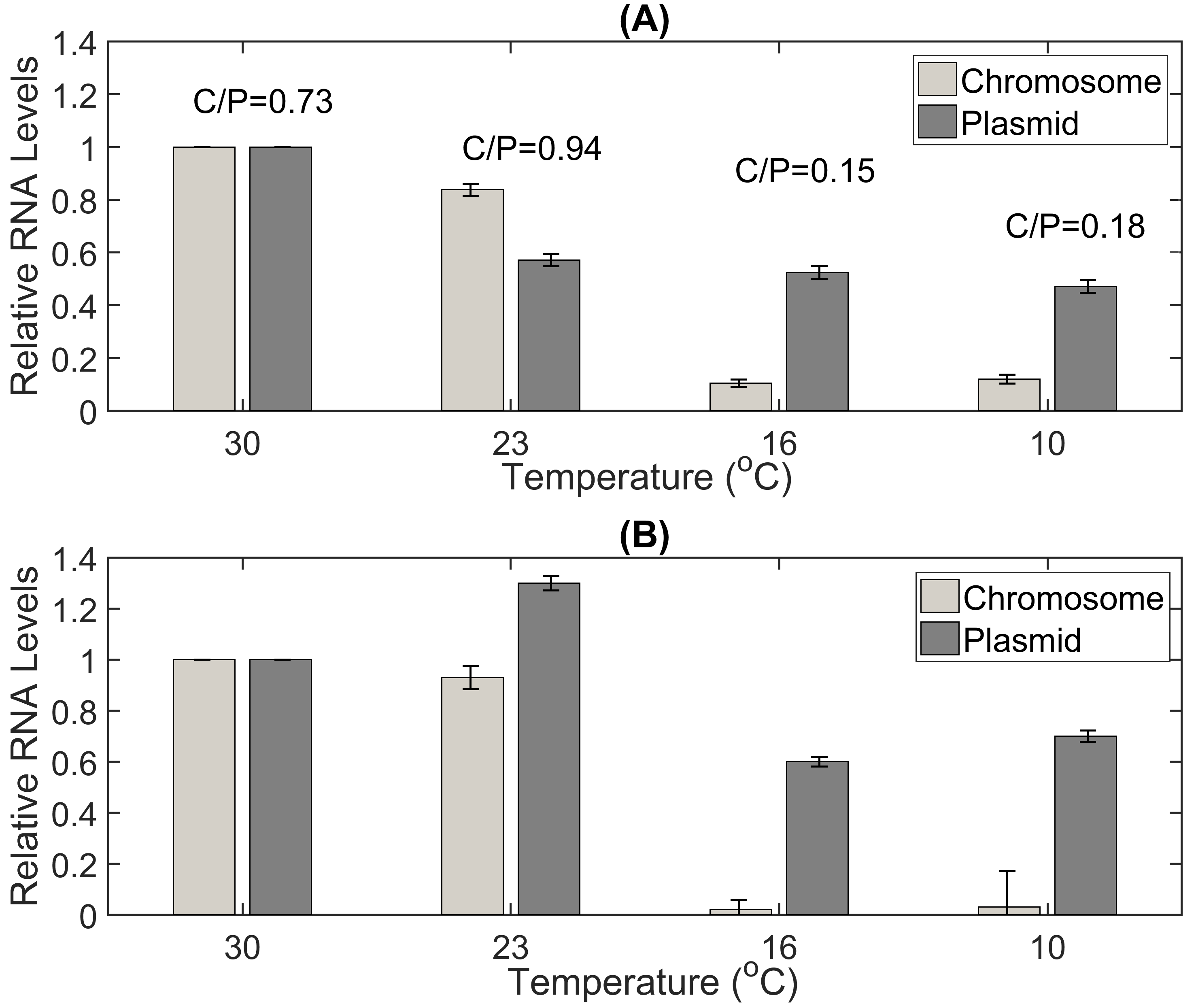


**Figure S6.** Mean relative RNA numbers in individual cells. Mean relative RNA numbers in individual cells subject to full induction (1 mM IPTG) at different temperatures relative to the control (30°C) as measured by (A) microscopy and by (B) RT-PCR. Cells carrying the chromosome (light gray) and plasmid (dark gray) construct were induced 1 hour prior to the measurements. RT-PCR (3 technical replicates) and microscopy measurements are relative to the 30 °C condition (thus removing the error from that point). Data presented as relative mean to the reference case with 90% confidence intervals obtained from a two-tailed Student’s t-test. Also shown is the ratio (C/P) between the integer-valued mean RNA numbers per cell between cells with the target gene chromosomally-integrated (C) and on a single-copy plasmid (P).


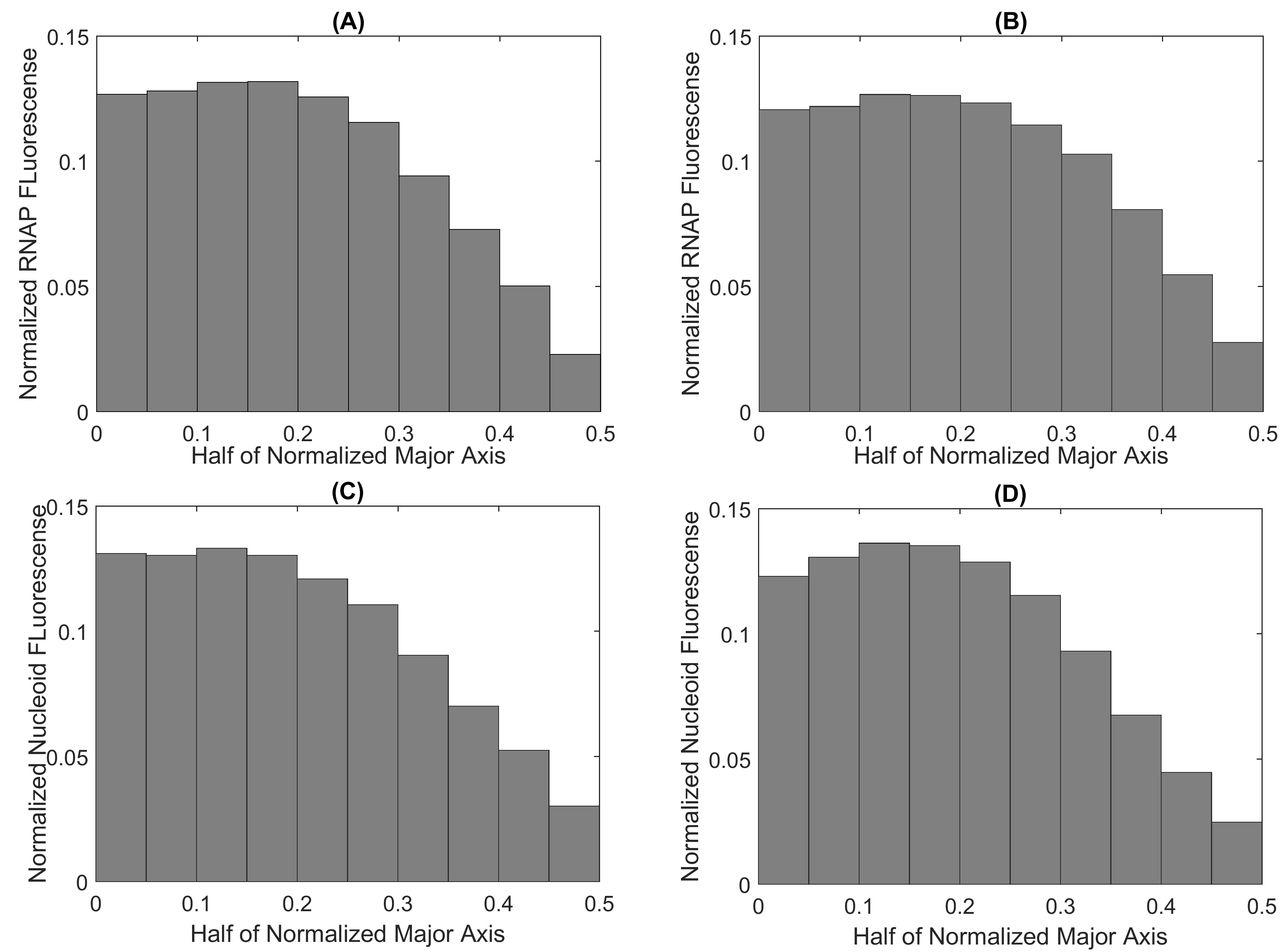


**Figure S7.** RNAP and nucleoid fluorescence along the major cell axis. RNAP fluorescence along the major cell axis (binned) as normalized by the total mean fluorescence of the cells (RL1314 strain). Measurements at (A) 30 °C from 614 cells and (B) 10 °C from 613 cells. A two-sample KS test comparing the spatial distributions at 10 °C and 30 °C fails to reject that they are from the same distribution (P value = 0.98). Also shown is the normalized average nucleoid ﬂuorescence intensity distribution along the normalized major axis of the cells as measured by DAPI staining. Measurements are from (C) 614 cells at 30 °C, and (D) 613 cells at 10 °C. A two-sample KS test comparing the spatial distributions at 10 °C and 30 °C fails to reject that they are from the same distribution (P value = 0.99). In all figures, in the x axis, ‘0’ corresponds to the cell center, while ‘0.5’ corresponds to both extremities (cells folded in half, with undefined poles). Cells were fixed with formaldehyde prior to imaging.


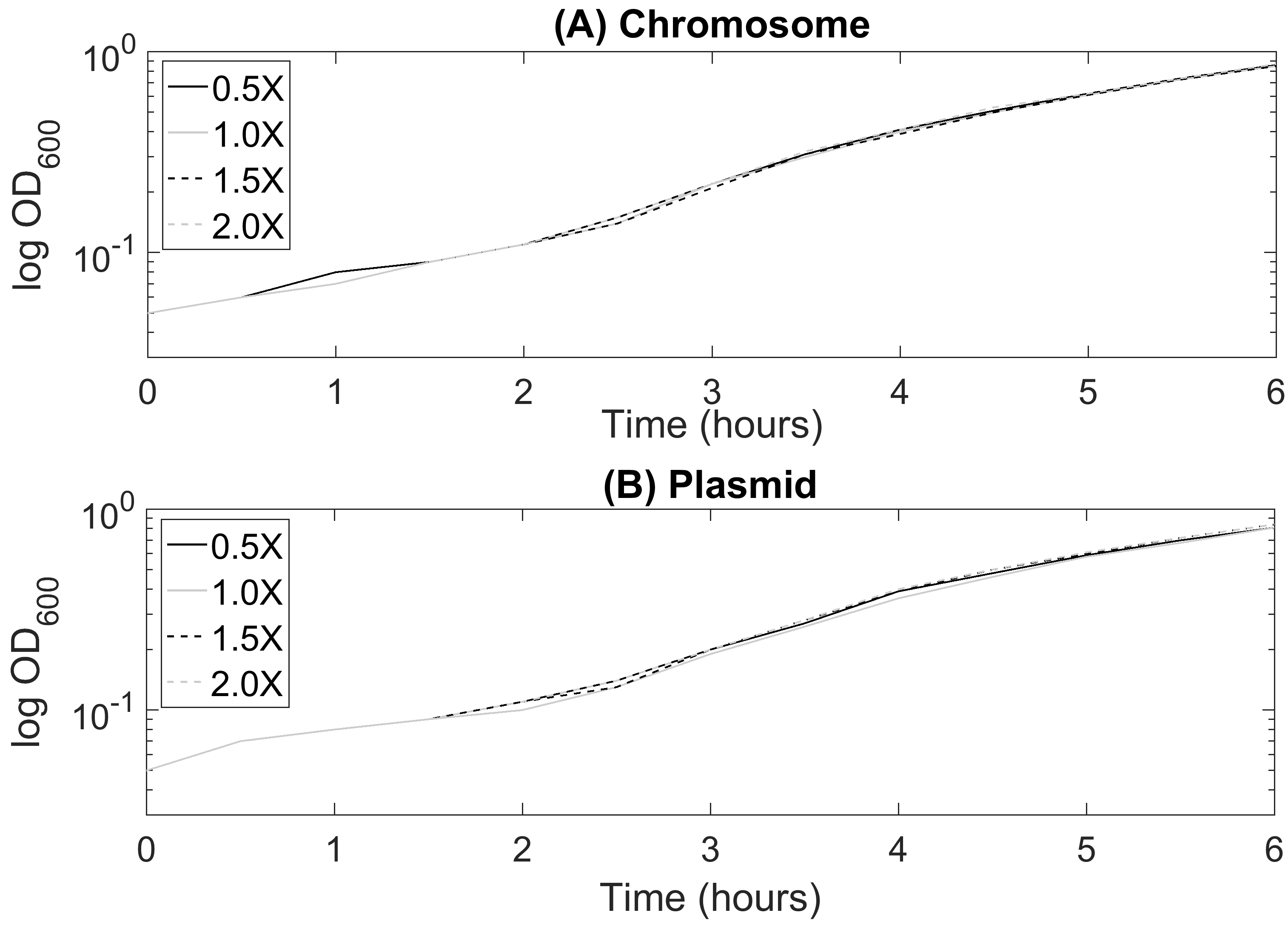


**Figure S8.** Cell growth curves. Growth curves (OD_600_, measured by an Ultrospec 10 Cell Density Meter, Amersham Bioscience) of cells carrying the (A) chromosome and (B) plasmid constructs at 30 °C. Cells grown overnight in LB medium at 30 °C with aeration of 250 rpm, and diluted into fresh 0.5X, 1X, 1.5X and 2X medium to an initial OD_600_ of 0.05. Next, they were incubated at 37 °C until reaching the mid-log phase (first 3 hours), and placed at 30 °C for the remaining 3 hours. The OD_600_ was measured every 30 minutes.


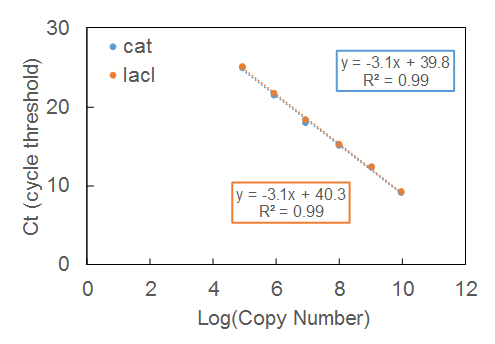


**Figure S9.** Standard curves for the *cat* and *lacI* genes. The curve was constructed using 10-fold serial dilutions of pCA24N-ligase, (ranging from 10^4^ to 10^9^ copies/μl). Each dilution was amplified by RT-qPCR, in triplicates, using primer sets specifics for *cat* and *lacI* genes. For each gene, the C_T_ values were plotted against the logarithm of their known initial copy number, and a standard curve was generated by a linear regression through these values.


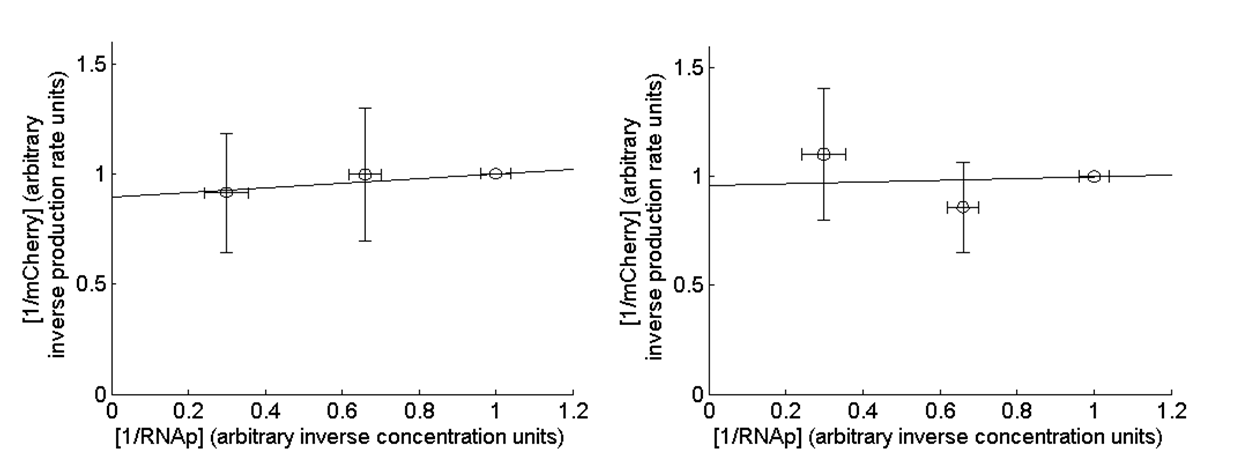


**Figure S10.** Lineweaver-Burk plot of the inverse of the relative production rate of mCherry from the P_lacO3O1_ promoter against the inverse of relative difference of RNAp concentrations for cells grown in three different media (1.0x, 1.5x, 2.0x M9-Gly) at 30°C. RNAp concentrations are presented relative to the RNAp concentration at 1.0x, from the difference of RNAp concentration at 0.5x (Table S7). Relative production rates were measured by RT-PCR with three technical replicates for each condition relative to 1.0x condition (Table S9). (Left) chromosome integrated construct. (Right) plasmid integrated construct. The linear relationship between the points for each relative production rate and its corresponding relative difference of RNAp concentration (circles) is visible in both constructs. A likelihood ratio test was performed to determine whether the small deviations from linearity are statistically signiﬁcant. In no construct was linearity rejected (P > 0.25 in both cases). Standard uncertainties are shown (horizontal and vertical error bars in each data point).

**Supplementary Tables**

**Table S1.** Mean number of tagged RNAs produced under the control of P_LacO3O1_ in cells lacking the target gene for MS2-GFP, and non-induced cells carrying the target gene (chromosome and plasmid constructs). Measurements were obtained 1 hour after the start of incubation of the cells in liquid culture at 30°C. Shown are the number of cells observed and their mean integer-valued RNA numbers per cell. In cells lacking the target gene, there is no coding ability of the RNA target for MS2-GFP and thus, in these, any ‘detected’ RNA is a false positive due to MS2-GFP. Measurements were conducted at 30 °C and at 10 °C.

| Condition | No. cells | Absolute mean RNA no. per cell |
| --- | --- | --- |
| No target gene (30 °C) | 612 | 0.015 |
| No target gene (10 °C) | 630 | 0.022 |
| Plasmid construct, no induction (30 °C) | 670 | 0.919 |
| Plasmid construct, no induction (10 °C) | 605 | 0.466 |
| Chromosome construct, no induction (30 °C) | 665 | 0.332 |
| Chromosome construct, no induction (10 °C) | 615 | 0.135 |

**Table S2.** Absolute mean half-life times of RNA molecules tagged with MS2-GFP in the two strains and temperature conditions, extracted from 1-hour long time series, with images taken every minute. Mean half-lives were obtained by fitting the intensity of each RNA over time with a decaying exponential function and then inferring the degradation rate of the RNA fluorescence intensity. Note that no tagged RNA was observed to ‘disappear’ during any measurement.

| Construct | Temperature (°C) | No. RNAs | Mean half-life (min) |
| --- | --- | --- | --- |
| Chromosome | 30 | 10 | 151.3 |
| Plasmid | 30 | 10 | 148.0 |
| Chromosome | 10 | 10 | 121.6 |
| Plasmid | 10 | 40 | 120.2 |

**Table S3.** P values of the KS tests comparing pairs of distributions of integer-valued RNA numbers per cell**.** Each distribution corresponds to a given induction level for the the induction of the targer gene of the plasmid construct (top table) and the chromossome construct (bottom table). In these tests, for P values smaller than 0.01, the null hypothesis that the two sets of data are from the same distribution is rejected. These tables are related to Figure 1 in main manuscript.

| Plasmid | | | | | |
| --- | --- | --- | --- | --- | --- |
|  | 50 µM | 100 µM | 250 µM | 500 µM | 1000 µM |
| 0 µM | 5.6×10^-27^ | 1.7×10^-27^ | 2.9×10^-28^ | 7.8×10^-33^ | 5.5×10^-42^ |
| 50 µM | - | 0.2276 | 8.0×10^-7^ | 3.5×10^-6^ | 7.9×10^-7^ |
| 100 µM | - | - | 0.001 | 0.018 | 3.4×10^-6^ |
| 250 µM | - | - | -- | 0.7641 | 0.015 |
| 500 µM | - | - | - | - | 0.206 |
| Chromosome | | | | | |
|  | 50 µM | 100 µM | 250 µM | 500 µM | 1000 µM |
| 0 µM | 2.7×10^-15^ | 3.1×10^-13^ | 2.8×10^-16^ | 2.1×10^-16^ | 2.7×10^-13^ |
| 50 µM | - | 0.956 | 0.906 | 0.040 | 0.190 |
| 100 µM | - | - | 0.893 | 0.225 | 0.730 |
| 250 µM | - | - | - | 0.190 | 0.845 |
| 500 µM | - | - | - | - | 0.917 |

**Table S4.** P values of the KS tests comparing the distributions of integer-valued RNA numbers per cell between temperature conditions for the plasmid construct and for the chromossome construct. In these tests, for P values smaller than 0.01, the null hypothesis that the two sets of data are from the same distribution is rejected. This table is related to Table 1 in main manuscript.

| Condition | Plasmid | Chromosome |
| --- | --- | --- |
| 30°C vs 27°C | 2×10^-4^ | 3.4×10^-7^ |
| 27°C vs 23°C | 3.7×10^-7^ | 1.7×10^-4^ |
| 23°C vs 20°C | 0.138 | 6.0×10^-19^ |
| 20 °C vs 16 °C | 1 | 0.008 |
| 16 °C vs 10°C | 0.58 | 1.000 |

**Table S5.** P values of the KS tests comparing the distributions of integer-valued RNA numbers per cell between the two constructs at the various temperatures. In these tests, for P values smaller than 0.01, the null hypothesis that the two sets of data are from the same distribution is rejected. This table is related to Table 1 in main manuscript.

| Condition | Plasmid vs Chromosome |
| --- | --- |
| 30 °C | 9.2×10^-24^ |
| 27 °C | 0.044 |
| 23 °C | 0.177 |
| 20 °C | 3.5×10^-14^ |
| 16 °C | 1.6×10^-28^ |
| 10 °C | 3.9×10^-12^ |

**Table S6.** P values of the KS tests comparing the distributions of the DAPI fluorescence levels in individual cells as a function of the conditions (0.5X, 1.0X, 1.5X and 2.0X), in RL1314 cells at 30 °C and 10 °C. In these tests, for P values smaller than 0.01, the null hypothesis that the two sets of data are from the same distribution is rejected. Cells were fixed with formaldehyde prior to imaging.

| 30 °C | | | |
| --- | --- | --- | --- |
| Media Richness | 0.5X | 1.0X | 1.5X |
| 1.0X | 0.655 | - | - |
| 1.5X | 0.310 | 0.162 | - |
| 2.0X | 0.697 | 0.964 | 0.182 |
| 10 °C | | | |
| Media Richness | 0.5X | 1.0X | 1.5X |
| 1.0X | 0.029 | - | - |
| 1.5X | 0.057 | 0.787 | - |
| 2.0X | 0.001 | 0.697 | 0.511 |

**Table S7.** Number of cells observed, mean and standard deviation of fluorescence intensity from RNAP-GFP in individual cells (arbitrary units, A.U.), absolute difference between mean RNAP-GFP intensity and the 0.5X condition, ratio between these values and the 1X control condition, and inverse of this value. Data from RL1314 cells at 30 °C and at 10 °C. This table is related to Table 2 in main manuscript. Cells were fixed with formaldehyde prior to imaging.

| Condition | No. cells | Mean RNAP fluorescence per cell (A.U.) | Absolute difference to 0.5X condition | Relative value to 1X condition | Inverse of the relative value to 1X condition |
| --- | --- | --- | --- | --- | --- |
| 30 °C |  |  |  |  |  |
| 0.5X media | 605 | 1.50±0.03 | 0 | - | - |
| 1.0X media | 614 | 1.55±0.03 | 0.05 | 1 | 1 |
| 1.5X media | 614 | 1.58±0.03 | 0.07 | 1.6 | 0.6 |
| 2.0X media | 612 | 1.66±0.05 | 0.16 | 3.2 | 0.3 |
| 10 °C |  |  |  |  |  |
| 0.5X media | 607 | 1.47±0.03 | 0 | - | - |
| 1.0X media | 613 | 1.52±0.03 | 0.05 | 1 | 1 |
| 1.5X media | 616 | 1.63±0.03 | 0.16 | 3.2 | 0.3 |
| 2.0X media | 603 | 1.78±0.03 | 0.31 | 6.2 | 0.2 |

**Table S8.** P values of the KS tests comparing the distributions of RNAP fluorescence levels in individual cells as a function of the conditions (0.5X, 1.0X, 1.5X and 2.0X), in RL1314 cells at 30 °C and 10 °C. In these tests, for P values smaller than 0.01, the null hypothesis that the two sets of data are from the same distribution is rejected. This table is related to Table 2 in main manuscript.

| 30 °C | | | |
| --- | --- | --- | --- |
| Media Richness | 0.5X | 1.0X | 1.5X |
| 1.0X | 0.009 | - | - |
| 1.5X | 6.4×10^-4^ | 0.728 | - |
| 2.0X | 9.0×10^-5^ | 0.313 | 0.253 |
| 10 °C | | | |
| Media Richness | 0.5X | 1.0X | 1.5X |
| 1.0X | 0.168 | - | - |
| 1.5X | 0.001 | 0.038 | - |
| 2.0X | 2.1×10^-14^ | 4.4×10^-10^ | 6.5×10^-6^ |

**Table S9.** Inverse of the RNA production rates in the 1.5X and 2.0X conditions relative to the 1X condition. Data from the chromossome integrated and the plasmid integrated constructs when cells are at 30 °C and at 10 °C. This table is related to Table 2 in main manuscript.

| Condition | 1X | 1.5X | 2.0X |
| --- | --- | --- | --- |
| 30 °C |  |  |  |
| Plasmid construct | 1 | 0.99 | 0.91 |
| Chromosome construct | 1 | 0.86 | 1.1 |
| 10 °C |  |  |  |
| Plasmid construct | 1 | 0.95 | 1.09 |
| Chromosome construct | 1 | 0.66 | 1.04 |

**Table S10.** Number of cells observed, along with the mean values of the absolute length (in µm) of the major and minor cell axes, and major and minor nucleoid axes at various temperatures. Also shown is the length of the major and minor axes of the nucleoid, relative to the cell major and minor axes lengths. Cells were fixed with formaldehyde prior to imaging.

| Measurements | 10 °C | 16 °C | 23 °C | 30 °C |
| --- | --- | --- | --- | --- |
| No. cells | 613 | 613 | 615 | 614 |
| Absolute Major Cell Axis | 3.79 | 3.54 | 3.14 | 3.00 |
| Absolute Minor Cell Axis | 1.19 | 1.20 | 1.13 | 1.08 |
| Absolute Major Nucleoid Axis | 2.47 | 2.19 | 1.99 | 1.77 |
| Absolute Minor Nucleoid Axis | 0.84 | 0.85 | 0.88 | 0.78 |
| Relative Major Nucleoid Axis | 0.65 | 0.62 | 0.64 | 0.59 |
| Relative Minor Nucleoid Axis | 0.71 | 0.71 | 0.78 | 0.73 |

**Table S11. Parameter values of the rate constants of transcription in model cells.** The value of k_1_ accounts for the expected RNA polymerase concentration in the cells. k_3_ is set to infinite (∞), as it is much faster than all other rate constants.

| Rate Constants | Parameter Value (s^-1^) | Reference |
| --- | --- | --- |
| k_ON_ | 0.011 | (1) |
| k_rep_ | 281 | (1) |
| k_1_ | 6469 | (1) |
| k_-1_ | 1 | (1) |
| k_2_ | 0.005 | (1) |
| k_3_ | ∞ | (1) |
| k_unlock_ | See Figure 6 | (21) |

**Table S12.** Estimated plasmid copy number, using absolute quantification. Shown are the average and the standard deviation of the triplicates results obtained by RT-qPCR.

| T (°C) | C_T_ | | Copies (μl^-1^) | | Absolute Plasmid Copy Number |
| --- | --- | --- | --- | --- | --- |
|  | *cat* | *lacI* | *cat* | *lacI* |  |
| 10 °C | 22.98 ± 0.03 | 23.34 ± 0.07 | 5.46 ± 0.02 | 5.47 ± 0.01 | 1.00 ± 0.00 |
| 30 °C | 23.00 ± 0.13 | 23.68 ± 0.04 | 5.46 ± 0.01 | 5.36 ± 0.04 | 1.02 ± 0.01 |

**Table S13. Bacterial strains used in this study.**

| Strain | Genotype | Source |
| --- | --- | --- |
| *E. coli* BW25993 | F-, Δ(araD-araB)567, λ-, rph-1, Δ(rhaD-rhaB)568, lacI^q^, hsdR514 | (57) |
| *E. coli* BW25993 ∆lacZ::MS2-BS | F-, Δ(araD-araB)567, λ-, rph-1, Δ(rhaD-rhaB)568, lacI^q^, hsdR514, ∆lacZ::MS2-BS | In this study |
| *E. coli* RL1314 | F-, λ-, IN(rrnD-rrnE)1, rph-1, rpoC::GFP-kan | (5) |

**Table S14. Bacterial plasmids used in this study.**

| Plasmid | Genotype | Source |
| --- | --- | --- |
| pBELOBAC11 | Ori2, CmR, laczα, P_T7_, P_SP6_ | GenBank Accession #: U51113 |
| pBELOBAC11- P_LacO3O1_-BS (Target Plasmid) | Ori2, CmR, P_LacO3O1_-BS | In this study |
| pZA25-GFP (Reporter Plasmid) | pSC101, KanR, P_ara_-MS2-GFP | (58) |
| pZE11-P_rham_-gyrAB-sfGFP (Gyrases) | Ori, P_rham_, AmpR | In this study |
| pCA24N-ligase | Ori, CmR, lacI^q^, P_T5-lac_ | (54, 55) |

**Table S15.** Number of cells observed, mean and squared coefficient of variation (CV^2^) of the absolute integer-valued RNA numbers per cell, in cells with the chromosome-integrated construct, when grown at 30 °C or 10 °C and subject to Novobiocin or Topotecan for 90 minutes. Also shown are the P values of the KS-test of statistical significance comparing the two pairs of distributions of RNA numbers at the two temperature conditions. For *p* values smaller than 0.01, the null hypothesis that the two sets of data are from the same distribution is rejected. This table is related to Figure 3 in the main manuscript.

| Temperature and Perturbation | No. cells | Mean integer-valued RNA no. per cell | CV^2^ | *P* value of the KS-test |
| --- | --- | --- | --- | --- |
| Novobiocin, 30 °C | 615 | 0.85 | 7.62 |  |
| Novobiocin, 10 °C | 615 | 0.25 | 8.7 | 0.004  (30 °C vs 10 °C) |
| Topotecan, 30 °C | 610 | 1.23 | 3.67 |  |
| Topotecan, 10 °C | 615 | 0.23 | 7.95 | 2.8×10^-11^  (30 °C vs 10 °C) |

**Table S16.** Mean integer-valued RNA numbers per cell as measured by microscopy and single RNA tagging by MS2-GFP in cells with a chromosomally-integrated or a plasmid-borne P_LacO3O1_ promoter, and in cells a the chromosomally-integrated native *lac*. Cells were at 10 °C or 30 °C, the target gene was either repressed (0 ***mM*** IPTG) or fully induced (1 mM IPTG), and Gyrase was or not overexpressed. Results are from 3 biological replicates. Since these exhibited no statistically significant differences, the results presented are composed of the data from the 3 biological replicates. Approximately 400 cells were analyzed in each condition. Mean RNA numbers per cell for the chromosomally-integrated and plasmid-borne P_LacO3O1_ promoters, at 0 and 1mM in both temperatures (without ***overexpressing*** ***Gyrase***), were extracted from Figure 2 of the main manuscript. Images were obtained 2 hours after starting incubation.

|  | 10 °C | 10 °C (Gyr) | 30 °C | 30 °C (Gyr) |
| --- | --- | --- | --- | --- |
|  | (Mean #RNA/cell) | (Mean #RNA/cell) | (Mean #RNA/cell) | (Mean #RNA/cell) |
| Chr - P_LacO3O1_ - 1mM IPTG | 0.33 | 0.84 | 2.07 | 2.05 |
| Chr - P_LacO3O1_ - 0mM IPTG | 0.14 | 0.29 | 0.33 | 0.34 |
| Chr - native *lac* - 1mM IPTG | 0.05 | 0.05 | 0.39 | 0.41 |
| Chr - native *lac* - 0mM IPTG | 0.03 | 0.03 | 0.22 | 0.23 |
| Plasmid - P_LacO3O1_ - 1mM IPTG | 1.39 | 1.46 | 3.11 | 3.31 |
| Plasmid - P_LacO3O1_ - 0mM IPTG | 0.47 | 0.50 | 0.92 | 0.97 |

**Supplementary References**

1. Chen, A. Y. & Liu, L. F. DNA topoisomerases: Essential enzymes and lethal targets. *Annu. Rev. Pharmacol. Toxicol.* **34,** 191–218 (1994).

2. Gellert, M., O’Dea, M. H., Itoh, T. & Tomizawa, J. I. Novobiocin and coumermycin inhibit DNA supercoiling catalyzed by DNA gyrase. *Proc. Natl. Acad. Sci. U. S. A.* **73,** 4474–4478 (1976).

3. Patel, K., Craig, S. B., Mcbride, M. G. & Palepu, N. R. Microbial inhibitory properties and stability of topotecan hydrochloride injection. *Am. J. Heal. Pharm.* **55,** 1584–1587 (1998).

4. de Boer, H. A., Bakker, A. J., Weyer, W. J. & Gruber, M. The role of energy-generating processes in the degradation of guanosine tetraphosphate, ppGpp, in *Escherichia coli*. *Biochim. Biophys. Acta (BBA)-Nucleic Acids Protein Synth.* **432,** 361–368 (1976).

5. Bratton, B. P., Mooney, R. A. & Weisshaar, J. C. Spatial distribution and diffusive motion of RNA polymerase in live *Escherichia coli*. *J. Bacteriol.* **193,** 5138–5146 (2011).

6. Lloyd-Price, J. *et al.* Dissecting the stochastic transcription initiation process in live *Escherichia coli*. *DNA Res.* **23,** 203–214 (2016).

7. Livak, K. J. & Schmittgen, T. D. Analysis of relative gene expression data using real-time quantitative PCR and the 2-DDCT method. *Methods* **25,** 402–408 (2001).

8. Santinha, J. *et al.* in *Biomedical Image Analysis and Mining Techniques for Improved Health Outcomes.* **i,** 71–99 (IGI Global, 2015).

9. Abdi, H. & Williams, L. . Principal Component Analysis. *Wiley Interdiscip. Rev. Comput. Stat.* **2,** 433–470 (2010).

10. Oliveira, S. M. D. *et al.* Increased cytoplasm viscosity hampers aggregate polar segregation in *Escherichia coli*. *Mol. Microbiol.* **99,** 686–699 (2016).

11. Mora, A. D., Vieira, P. M., Manivannan, A. & Fonseca, J. M. Automated drusen detection in retinal images using analytical modelling algorithms. *Biomed. Eng. Online* **10,** 59 (2011).

12. Häkkinen, A., Muthukrishnan, A.-B., Mora, A., Fonseca, J. M. & Ribeiro, A. S. CellAging: a tool to study segregation and partitioning in division in cell lineages of *Escherichia coli*. *Bioinformatics* **29,** 1708–1709 (2013).

13. Golding, I., Paulsson, J., Zawilski, S. M. & Cox, E. C. Real-time kinetics of gene activity in individual bacteria. *Cell* **123,** 1025–1036 (2005).

14. Golding, I. & Cox, E. C. RNA dynamics in live *Escherichia coli* cells. *Proc. Natl. Acad. Sci. U. S. A.* **101,** 11310–11315 (2004).

15. Mäkelä, J., Lloyd-Price, J., Yli-Harja, O. & Ribeiro, A. S. Stochastic sequence-level model of coupled transcription and translation in prokaryotes. *BMC Bioinformatics* **12,** 121 (2011).

16. Kandavalli, V. K., Tran, H. & Ribeiro, A. S. Effects of σ factor competition are promoter initiation kinetics dependent. *Biochim. Biophys. Acta (BBA)- Gene Regul. Mech.* **1859,** 1281–1288 (2016).

17. Muthukrishnan, A. B., Martikainen, A., Neeli-Venkata, R. & Ribeiro, A. S. *In vivo* transcription kinetics of a synthetic gene uninvolved in stress-response pathways in stressed *Escherichia coli* cells. *PLoS One* **9,** e109005 (2014).

18. Tran, H., Oliveira, S. M. D., Goncalves, N. & Ribeiro, A. S. Kinetics of the cellular intake of a gene expression inducer at high concentrations. *Mol. Biosyst.* 11, 2579–2587 (2015).

19. Chong, S., Chen, C., Ge, H. & Xie, X. S. Mechanism of Transcriptional Bursting in Bacteria. *Cell* **158,** 314–326 (2014).

20. DeHaseth, P. L., Lohman, T. M., Burgess, R. R. & Record, M. T. Nonspecific interactions of *Escherichia coli* RNA polymerase with native and denatured DNA: differences in the binding behavior of core and holoenzyme. *Biochemistry* **17,** 1612–1622 (1978).

21. Saecker, R. M., Record, M. T. & DeHaseth, P. L. Mechanism of Bacterial Transcription Initiation: RNA Polymerase - Promoter Binding, Isomerization to Initiation-Competent Open Complexes, and Initiation of RNA Synthesis. *J. Mol. Biol.* **412,** 754–771 (2011).

22. McClure, W. R. Mechanism and control of transcription initiation in prokaryotes. *Annu. Rev. Biochem.* **54,** 171–204 (1985).

23. Lutz, R. & Bujard, H. Independent and tight regulation of transcriptional units in *Escherichia coli* via the LacR/O, the TetR/O and AraC/I1-I 2 regulatory elements. *Nucleic Acids Res.* **25,** 1203–1210 (1997).

24. DeHaseth, P. L., Zupancic, M. L. & Record, M. T. RNA polymerase-promoter interactions: The comings and goings of RNA polymerase. *J. Bacteriol.* **180,** 3019–3025 (1998).

25. Peccoud, J. & Ycart, B. Markovian modelling of gene product synthesis. *Theor. Popul. Biol.* **48,** 222–234 (1995).

26. Bertrand-Burggraf, E., Lefèvre, J. F. & Daune, M. A new experimental approach for studying the association between RNA polymerase and the tet promoter of pBR322. *Nucleic Acids Res.* **12,** 1697–1706 (1984).

27. Buc, H. & McClure, W. R. Kinetics of open complex formation between *Escherichia coli* RNA polymerase and the lacUV5 promoter. Evidence for a sequential mechanism involving three steps. *Biochemistry* **24,** 2712–2723 (1985).

28. Chamberlin, M. J. The selectivity of transcription. *Annu. Rev. Biochem.* **43,** 721–775 (1974).

29. McClure, W. R. Rate-limiting steps in RNA chain initiation. *Proc. Natl. Acad. Sci. United States Am.* **77,** 5634–5638 (1980).

30. Hsu, L. Monitoring abortive initiation. *Methods* **47,** 25–36 (2009).

31. Hsu, L. M. Promoter clearance and escape in prokaryotes. *Biochim. Biophys. Acta - Gene Struct. Expr.* **1577,** 191–207 (2002).

32. Bai, L., Santangelo, T. J. & Wang, M. D. Single-Molecule Analysis of RNA Polymerase Transcription. *Annu. Rev. Biophys. Biomol. Struct.* **35,** 343–360 (2006).

33. Harden, T. T. *et al.* Bacterial RNA polymerase can retain σ70 throughout transcription. *Proc. Natl. Acad. Sci. U. S. A.* **113,** 602–607 (2016).

34. Mooney, R. A., Darst, S. A. & Landick, R. Sigma and RNA polymerase: An on-again, off-again relationship? *Mol. Cell* **20,** 335–345 (2005).

35. Raffaelle, M., Kanin, E. I., Vogt, J., Burgess, R. R. & Ansari, A. Z. Holoenzyme switching and stochastic release of sigma factors from RNA polymerase *in vivo*. *Mol. Cell* **20,** 357–366 (2005).

36. Epshtein, V. & Nudler, E. Cooperation Between RNA Polymerase Molecules in Transcription Elongation. *Science* **300,** 801–805 (2003).

37. Erie, D. A., Hajiseyedjavadi, O., Young, M. C. & von Hippel, P. H. Multiple RNA polymerase conformations and GreA: control of the fidelity of transcription. *Science* **262,** 867–873 (1993).

38. Greive, S. J. & von Hippel, P. H. Thinking quantitatively about transcriptional regulation. *Nat. Rev. Mol. Cell Biol.* **6,** 221–232 (2005).

39. Herbert, K. M. *et al.* Sequence-Resolved Detection of Pausing by Single RNA Polymerase Molecules. *Cell* **125,** 1083–1094 (2006).

40. Proshkin, S., Rahmouni, A. R., Mironov, A. & Nudler, E. Cooperation between translating ribosomes and RNA polymerase in transcription elongation. *Science* **328,** 504–508 (2010).

41. Mannerstrom, H., Yli-Harja, O. & Ribeiro, A. S. Inference of kinetic parameters of delayed stochastic models of gene expression using a Markov chain approximation. *Eurasip J. Bioinforma. Syst. Biol.* **1,** 572876 (2011).

42. Lloyd-Price, J., Gupta, A. & Ribeiro, A. S. SGNS2: A Compartmentalized Stochastic Chemical Kinetics Simulator for Dynamic Cell Populations. *Bioinformatics* **28,** 3004–3005 (2012).

43. Gillespie, D. T. Exact Stochastic Simulation of Coupled Chemical Reactions. *J. Phys. Chem.* **81,** 2340–2361 (1977).

44. Roussel, M. R. & Zhu, R. Validation of an algorithm for delay stochastic simulation of transcription and translation in prokaryotic gene expression. *Phys. Biol.* **3,** 274–284 (2006).

45. Lineweaver, H. & Burk, D. The determination of enzyme dissociation constants. *J. Am. Chem. Soc.* **56,** 658–666 (1934).

46. Cabrera, J. E. & Jin, D. J. Active transcription of rRNA operons is a driving force for the distribution of RNA polymerase in bacteria: Effect of extrachromosomal copies of rrnB on the *in vivo* localization of RNA polymerase. *J. Bacteriol.* **188,** 4007–4014 (2006).

47. Krystek, M. & Anton, M. A weighted total least-squares algorithm for fitting a straight line. *Meas. Sci. Technol.* **18,** 3438–3442 (2007).

48. Chen, H., Shiroguchi, K., Ge, H. & Xie, X. S. Genome-wide study of mRNA degradation and transcript elongation in *Escherichia coli*. *Mol. Syst. Biol.* **11,** 781 (2015).

49. Fieller, E. C. Some Problems in Interval Estimation. *J. R. Stat. Soc. Ser. B* **16,** 175–185 (1954).

50. Motulsky, H. *Intuitive Biostatistics: A Nonmathematical Guide to Statistical Thinking*. (1995).

51. Cooper, S. & Keasling, J. Cycle specific replication of chromosomal and F-plasmid origins. *FEMS Microbiol. Lett.* **163,** 217–222 (1998).

52. Keasling, J. D., Palsson, B. O. & Cooper, S. Cell-cycle-specific F plasmid replication: Regulation by cell size control of initiation. *J. Bacteriol.* **173,** 2673–2680 (1991).

53. Lee, C., Kim, J., Shin, S. G. & Hwang, S. Absolute and relative QPCR quantification of plasmid copy number in *Escherichia coli*. *J. Biotechnol.* **123,** 273–280 (2006).

54. Kitagawa, M. *et al.* Complete set of ORF clones of *Escherichia coli* ASKA library (A complete set of *E. coli* K-12 ORF archive): unique resources for biological research. *DNA Res.* **12,** 291–299 (2005).

55. Wilson, R. H. *et al.* Engineered DNA ligases with improved activities *in vitro*. *Protein Eng. Des. Sel.* **26,** 471–478 (2013).

56. Whelan, J. A., Russell, N. B. & Whelan, M. A. A method for the absolute quantification of cDNA using real-time PCR. *J. Immunol. Methods* **278,** 261–269 (2003).

57. Datsenko, K. & Wanner, B. One-step inactivation of chromosomal genes in *Escherichia coli* K-12 using PCR products. *Proc. Natl. Acad. Sci. U. S. A.* **97,** 6640–5 (2000).

58. Nevo-Dinur, K., Nussbaum-Shochat, A., Ben-Yehuda, S. & Amster-Choder, O. Translation-independent localization of mRNA in *E. coli*. *Science* **331,** 1081–1084 (2011).
